# Supplementary figures and images for: Major sex differences in allele frequencies for X chromosomal variants in both the 1000 Genomes Project and gnomAD
Source: PLoS Genet. 2022 May 31;18(5):e1010231. doi: 10.1371/journal.pgen.1010231 (PMC9187127; doi:10.1371/journal.pgen.1010231)

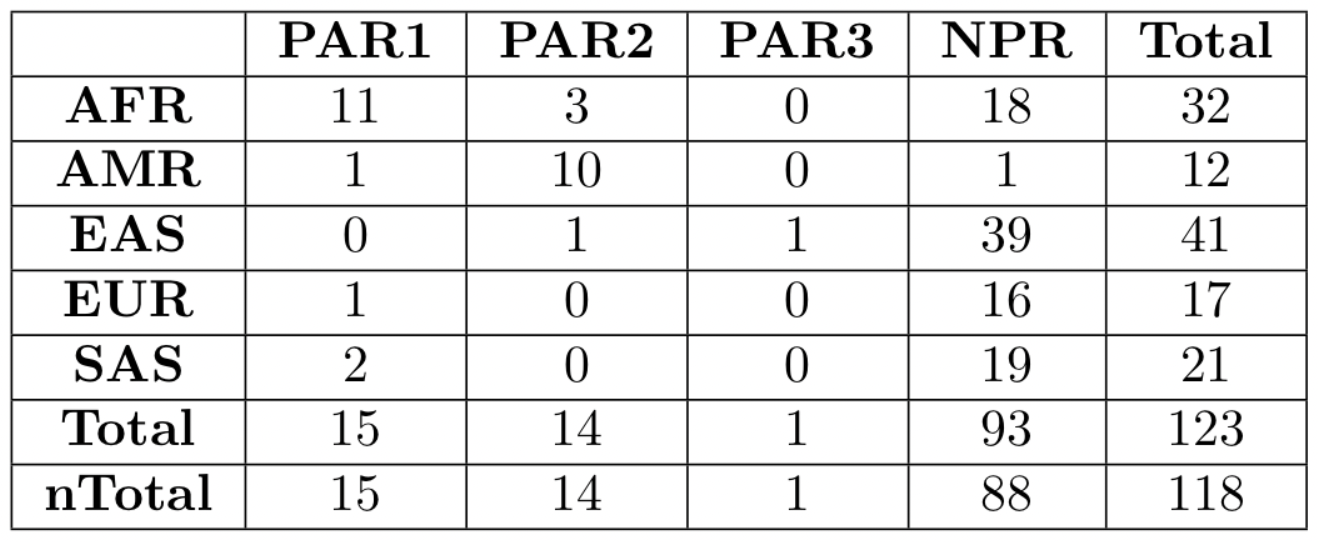

Supplement: S2 Table — The numbers of SNPs with genome-wide significant sdMAF in the superpopulation-specific analysis but not in the ALL analysis, stratified by the superpopulations and regions. The nTotal is the non-overlapping total, stratified by the four regions. (PNG) [file pgen.1010231.s002.png]

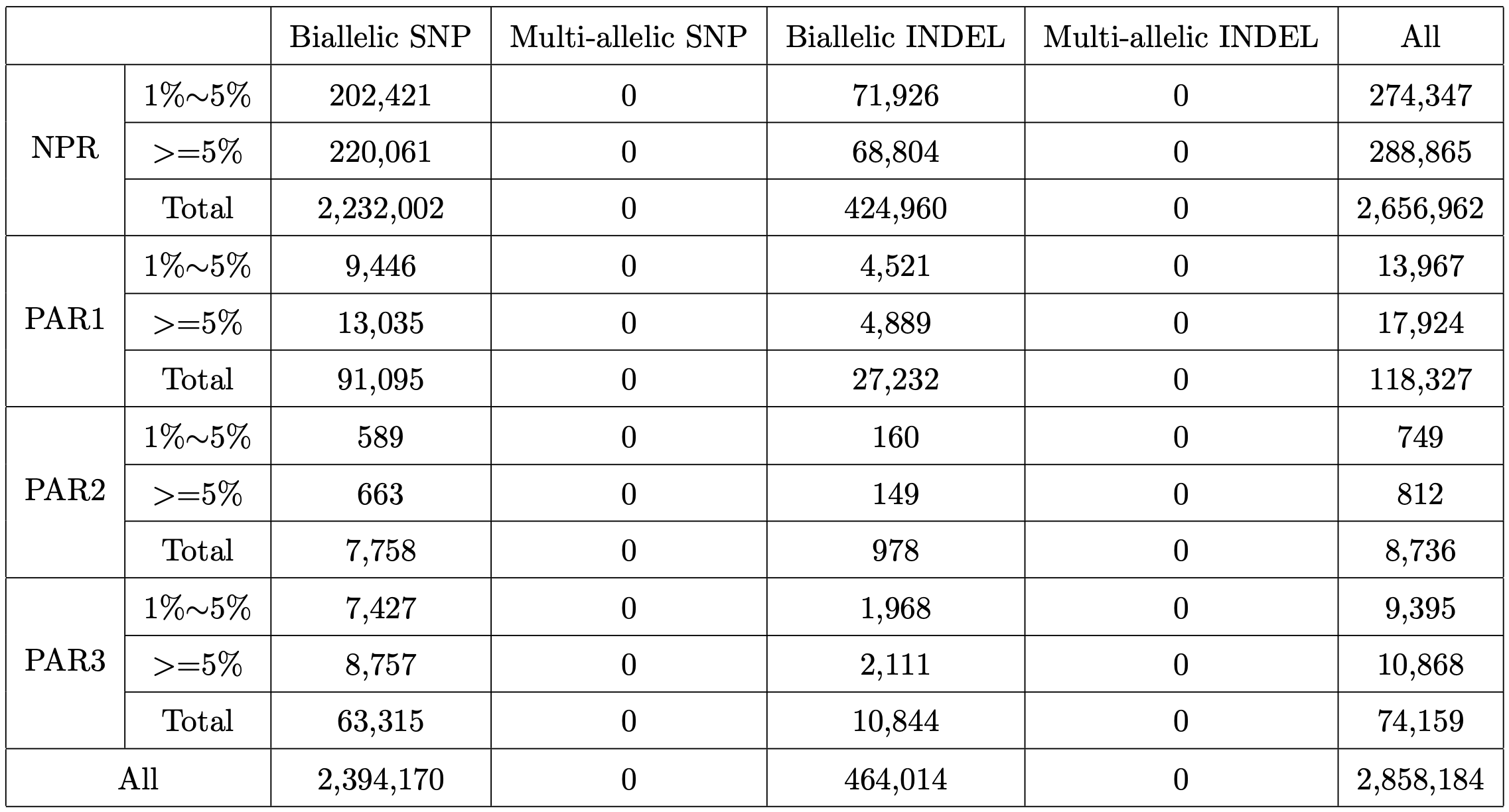

Supplement: S3 Table — See S19 Fig for the analytical pipeline of the SNP selection. (PNG) [file pgen.1010231.s003.png]

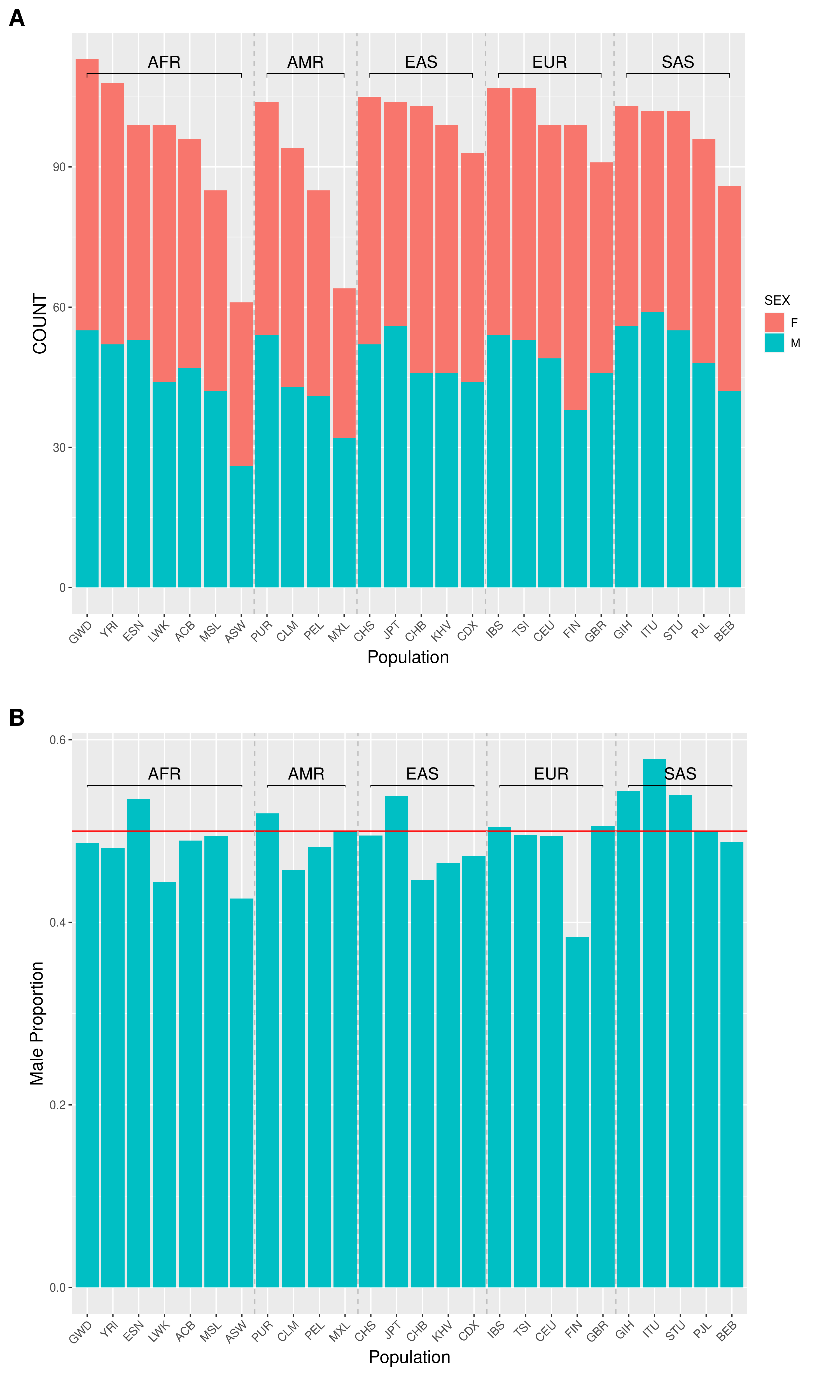

Supplement: S1 Fig — The populations are first ordered by the 5 super-populations alphabetically, and then by the total counts within each super-population. A: counts; B: the corresponding proportion of males. The red horizontal line represents 0.5. (TIFF) [file pgen.1010231.s005.tiff]

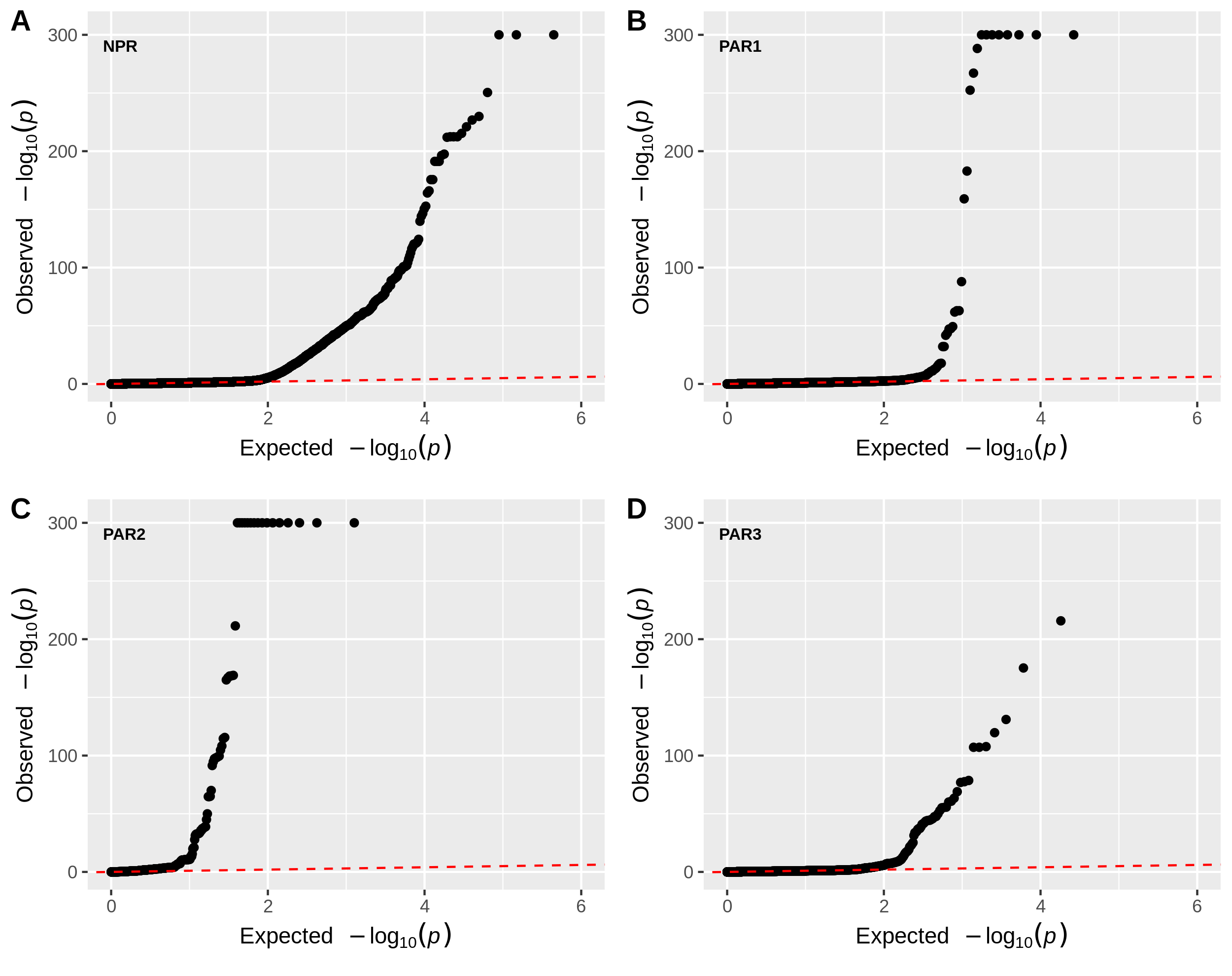

Supplement: S2 Fig — Results of bi-allelic SNPs with global MAF ≥5% are shown separately by region, A: NPR; B: PAR1, C: PAR2; D: PAR3. For better visualization p-values < 1e-300 are plotted as 1e-300 (300 on -log10 scale). The red dashed line represents the line of equality. The corresponding Manhattan plots are in Fig 2 (across the whole X chromosome) and Figs 4, 5 and 6 for PAR1, PAR2 and PAR3, respectively. (TIFF) [file pgen.1010231.s006.tiff]

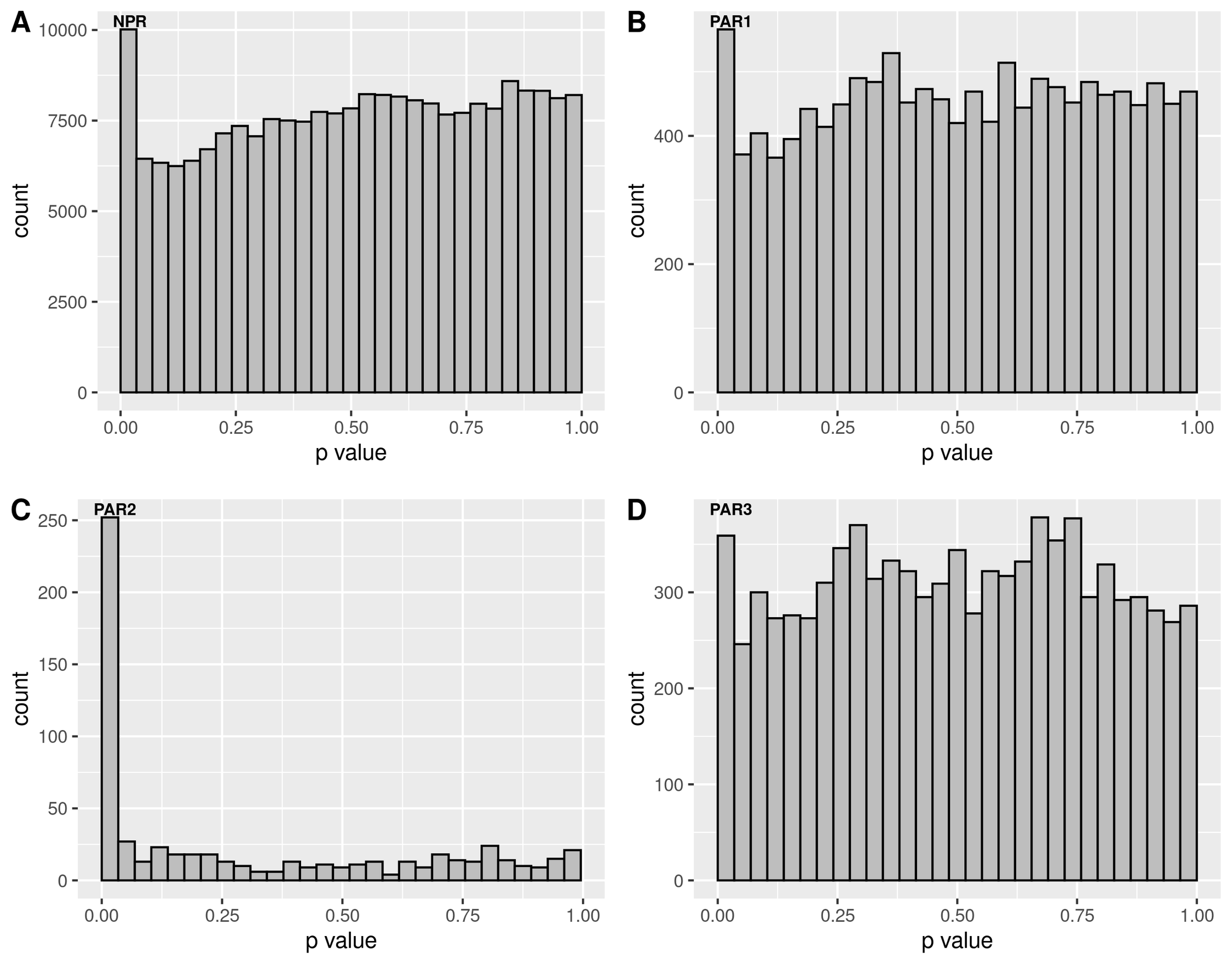

Supplement: S3 Fig — Results of bi-allelic SNPs with global MAF≥5% are shown separately by region, A: NPR; B: PAR1, C: PAR2; D: PAR3. (TIF) [file pgen.1010231.s007.tif]

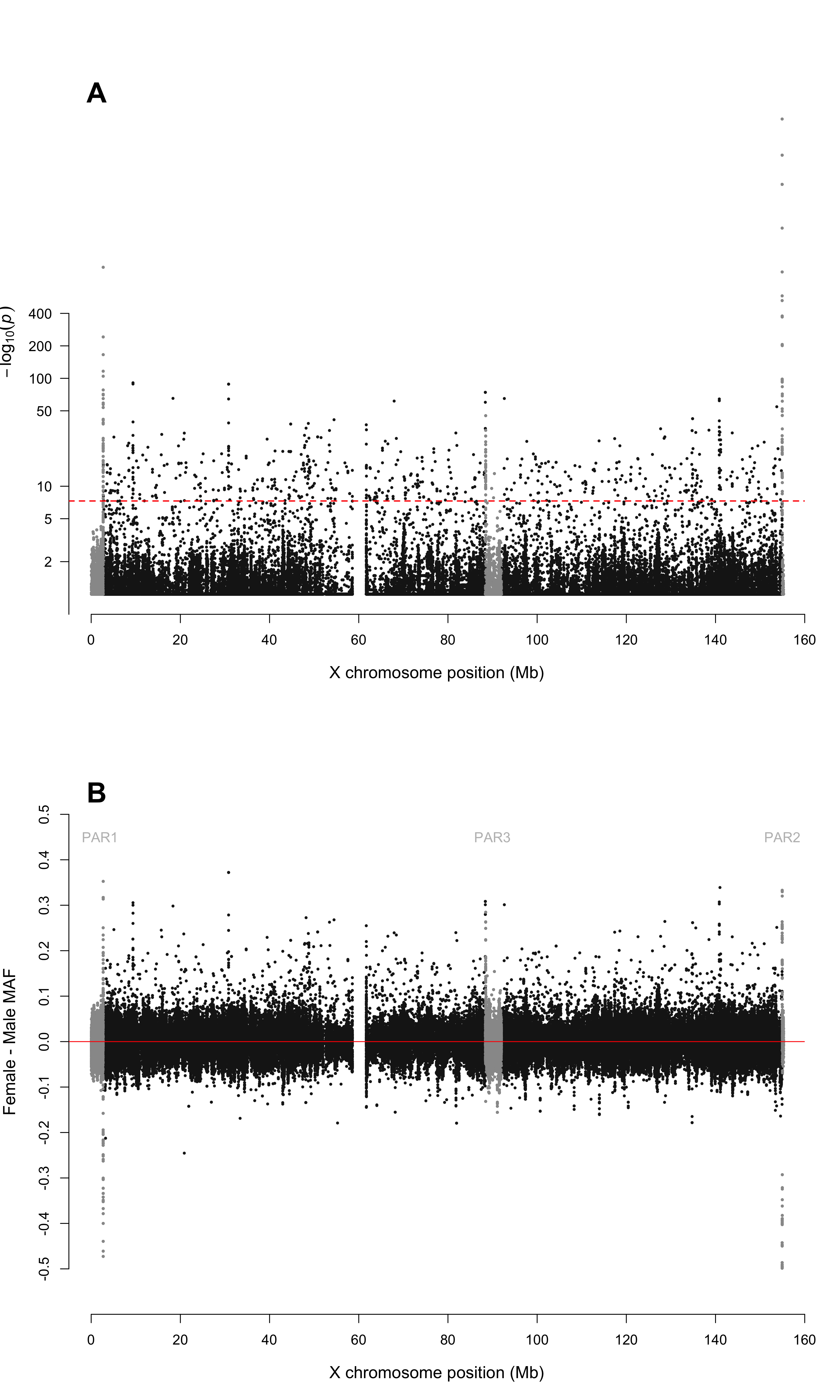

Supplement: S4 Fig — A: sdMAF p-values for bi-allelic SNPs with MAF ≥5% in superpopulation AFR presumed to be of high quality. SNPs in the PAR1, PAR2 and PAR3 regions are plotted in grey, with PAR3 located around 90 Mb. Y-axis is -log10(sdMAF p-values) and p-values >0.1 are plotted as 0.1 (1 on -log10 scale) for better visualization. The dashed red line represents 5e-8 (7.3 on the -log10 scale). B: Female—Male sdMAF for the same SNPs in part A. (TIFF) [file pgen.1010231.s008.tiff]

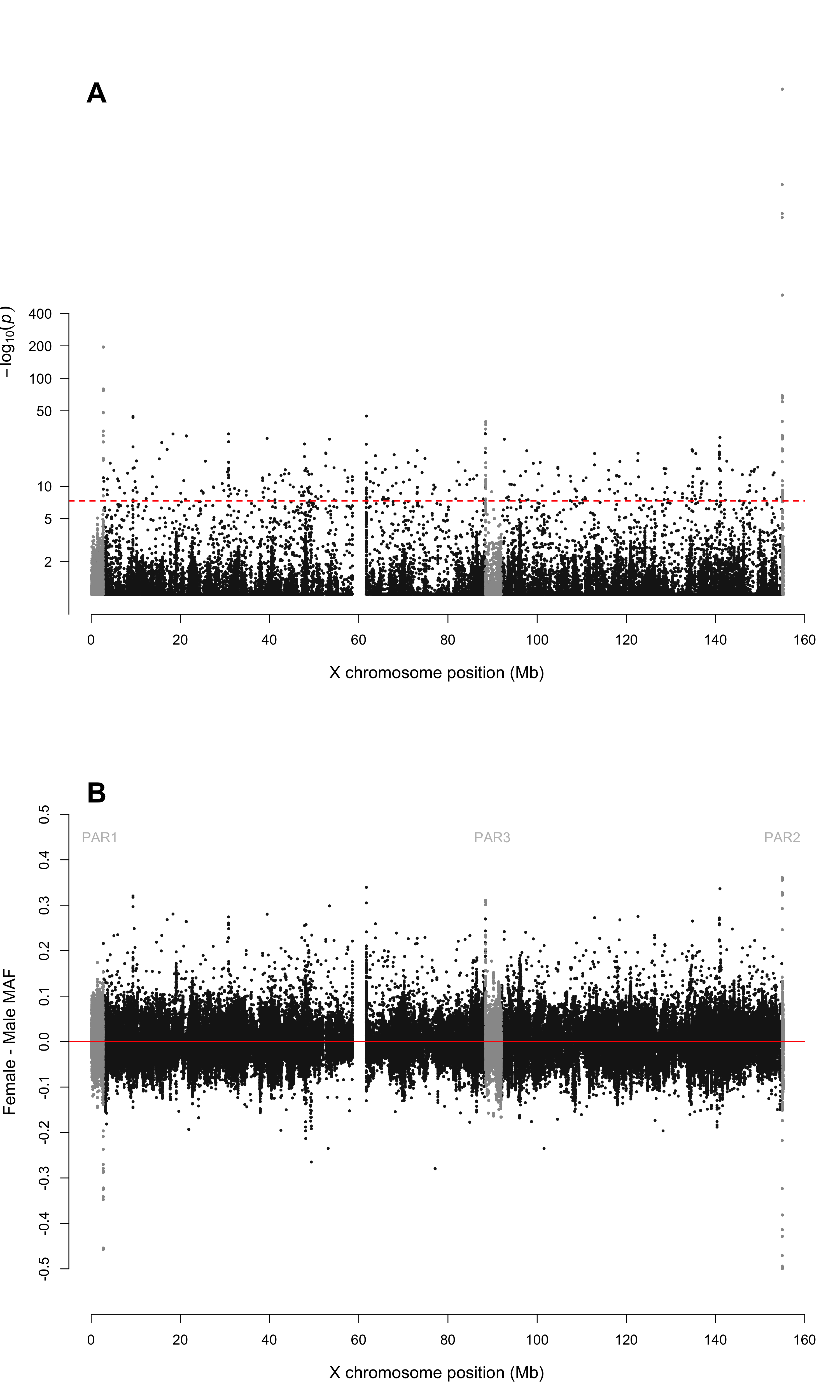

Supplement: S5 Fig — A: sdMAF p-values for bi-allelic SNPs with MAF ≥5% in superpopulation AMR presumed to be of high quality. SNPs in the PAR1, PAR2 and PAR3 regions are plotted in grey, with PAR3 located around 90 Mb. Y-axis is -log10(sdMAF p-values) and p-values >0.1 are plotted as 0.1 (1 on -log10 scale) for better visualization. The dashed red line represents 5e-8 (7.3 on the -log10 scale). B: Female—Male sdMAF for the same SNPs in part A. (TIFF) [file pgen.1010231.s009.tiff]

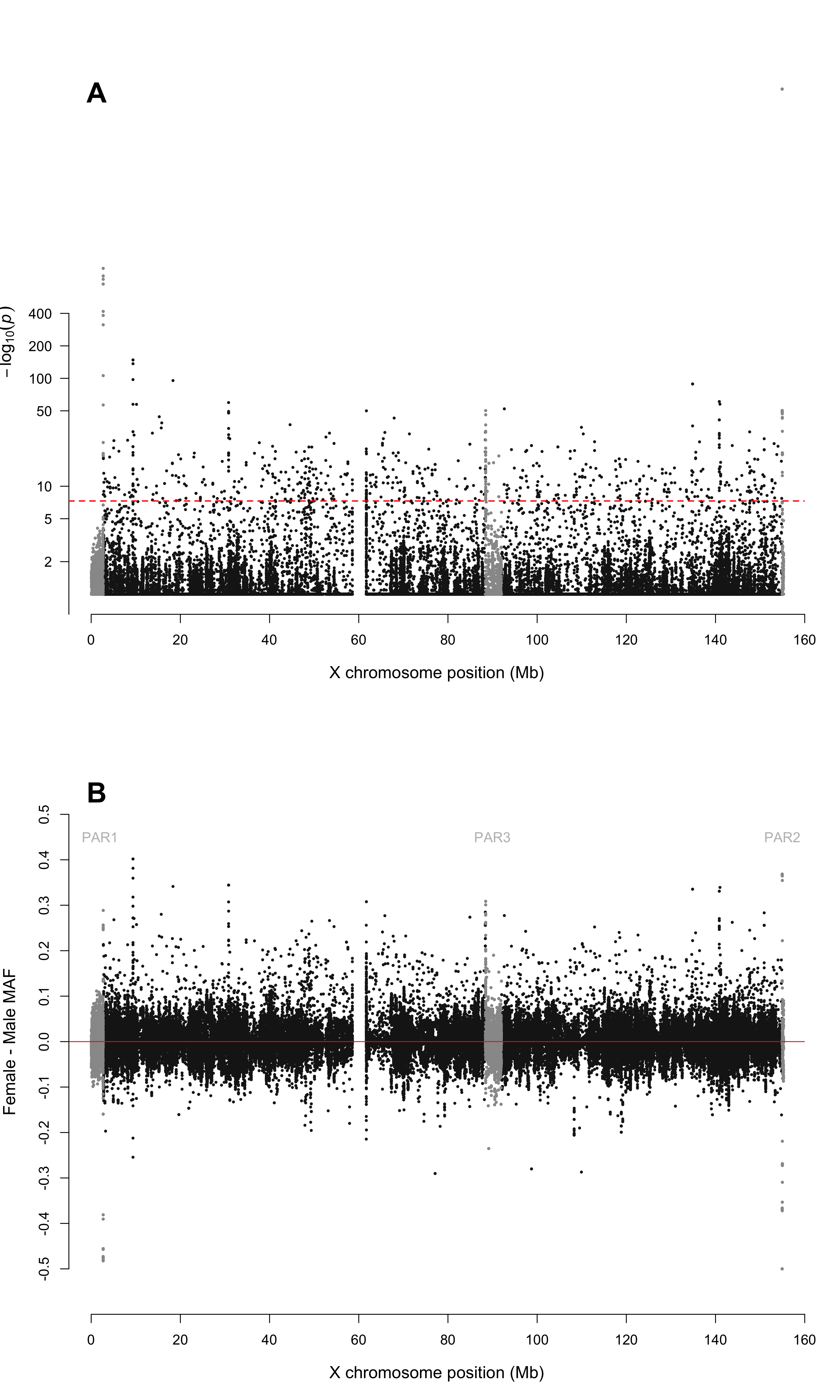

Supplement: S6 Fig — A: sdMAF p-values for bi-allelic SNPs with MAF ≥5% in superpopulation EAS presumed to be of high quality. SNPs in the PAR1, PAR2 and PAR3 regions are plotted in grey, with PAR3 located around 90 Mb. Y-axis is -log10(sdMAF p-values) and p-values >0.1 are plotted as 0.1 (1 on -log10 scale) for better visualization. The dashed red line represents 5e-8 (7.3 on the -log10 scale). B: Female—Male sdMAF for the same SNPs in part A. (TIFF) [file pgen.1010231.s010.tiff]

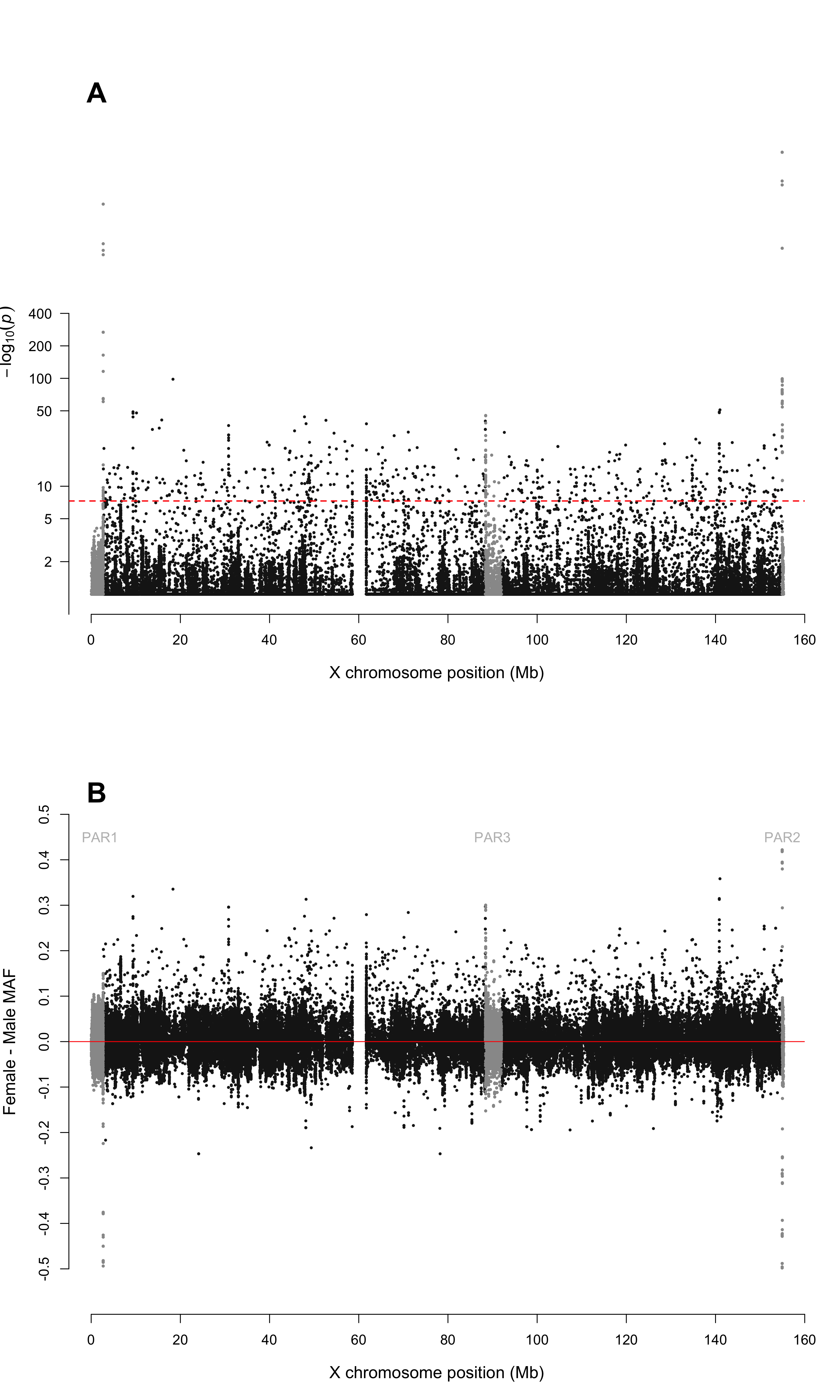

Supplement: S7 Fig — A: sdMAF p-values for bi-allelic SNPs with MAF ≥5% in superpopulation EUR presumed to be of high quality. SNPs in the PAR1, PAR2 and PAR3 regions are plotted in grey, with PAR3 located around 90 Mb. Y-axis is -log10(sdMAF p-values) and p-values >0.1 are plotted as 0.1 (1 on -log10 scale) for better visualization. The dashed red line represents 5e-8 (7.3 on the -log10 scale). B: Female—Male sdMAF for the same SNPs in part A. (TIFF) [file pgen.1010231.s011.tiff]

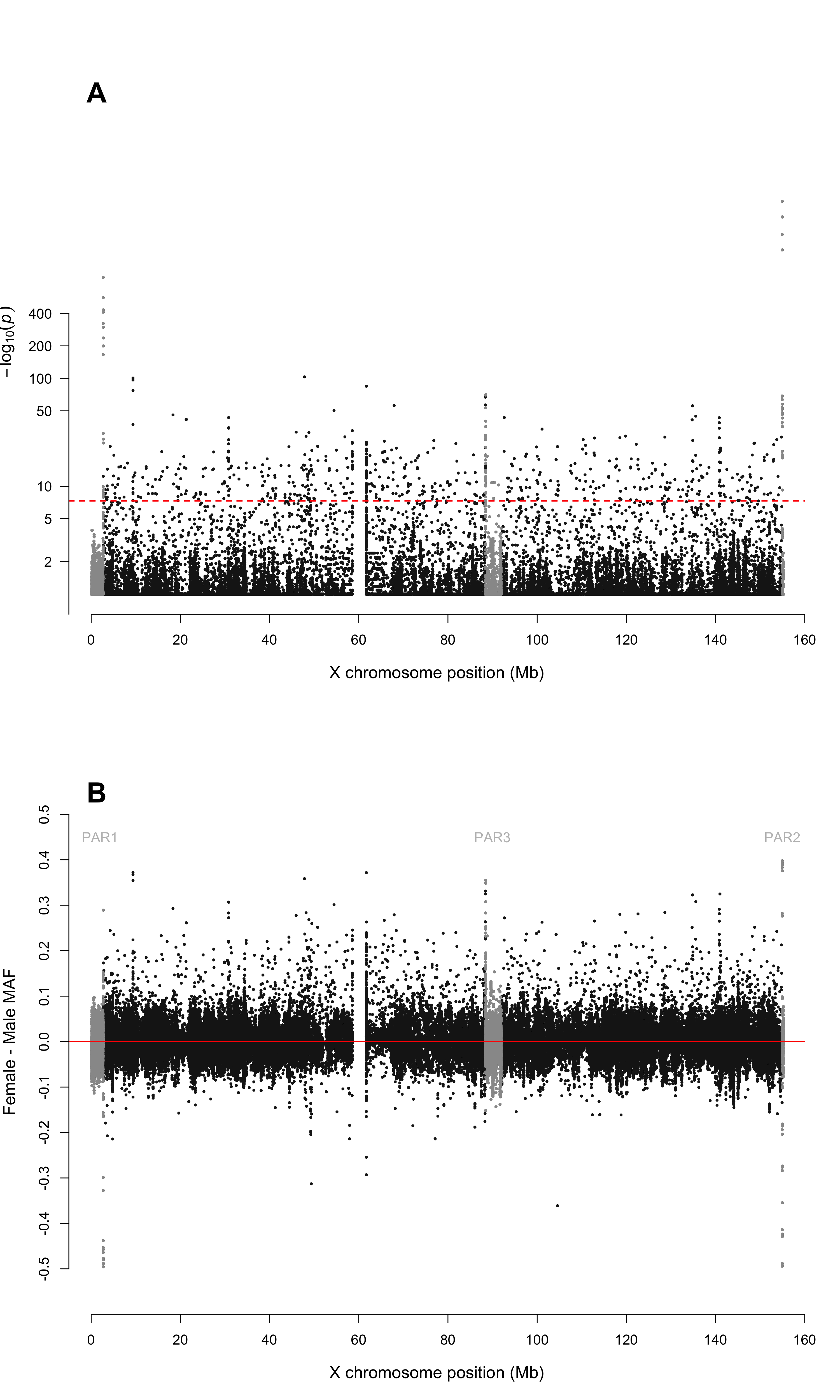

Supplement: S8 Fig — A: sdMAF p-values for bi-allelic SNPs with MAF ≥5% in superpopulation SAS presumed to be of high quality. SNPs in the PAR1, PAR2 and PAR3 regions are plotted in grey, with PAR3 located around 90 Mb. Y-axis is -log10(sdMAF p-values) and p-values >0.1 are plotted as 0.1 (1 on -log10 scale) for better visualization. The dashed red line represents 5e-8 (7.3 on the -log10 scale). B: Female—Male sdMAF for the same SNPs in part A. (TIFF) [file pgen.1010231.s012.tiff]

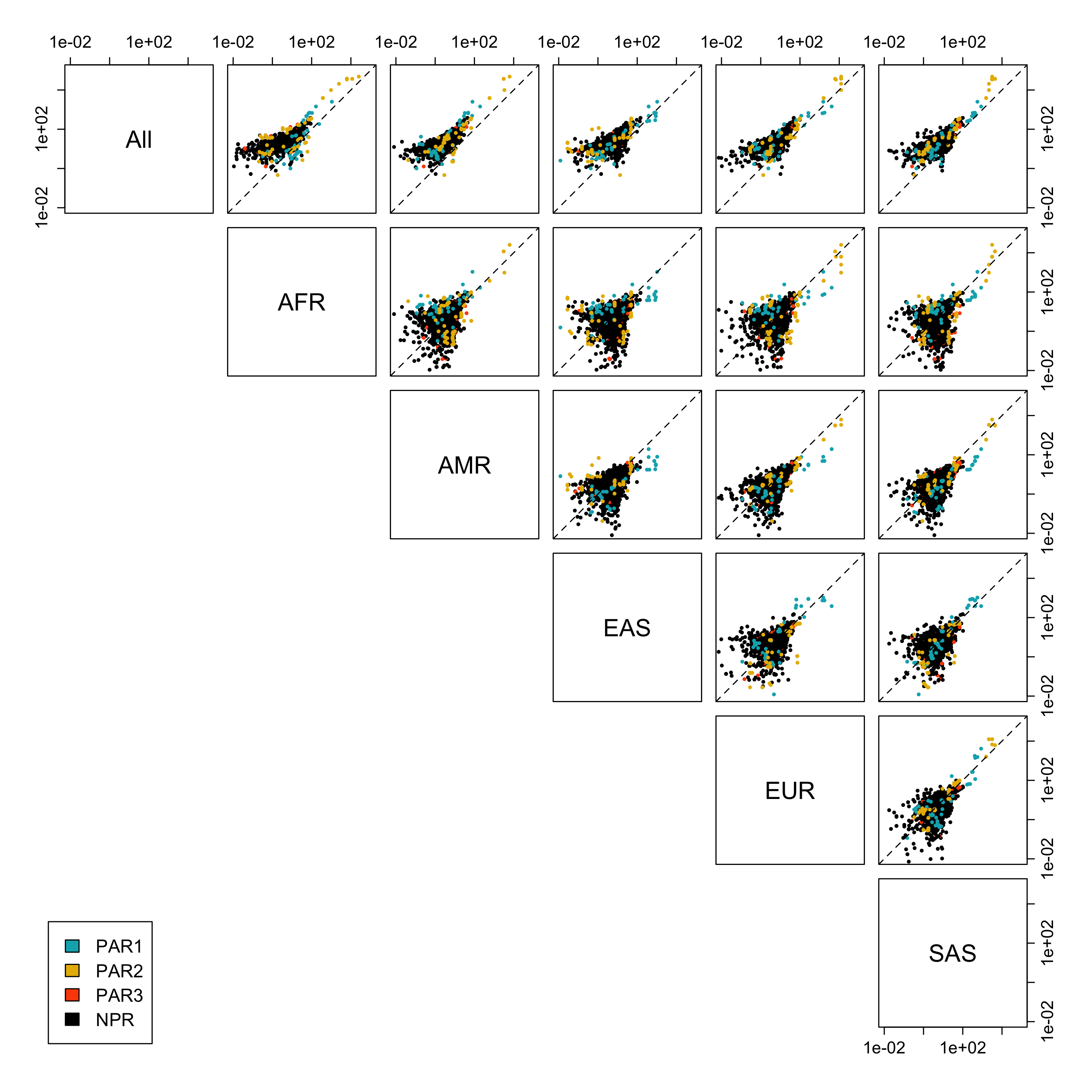

Supplement: S9 Fig — The bi-alleic SNPs shown are the ones with global MAF ≥5% in the combined ALL sample, polymorphic in each of the five superpopulations, and genome-wide significant in at least one of the six sdMAF analyses (i.e. in ALL or any of the five superpopulations). Both X-axis and Y-axis are -log10(sdMAF p-values) and log-scaled for better visualization. The dashed line is the main diagonal line. Each dot is colored based on the position of the corresponding SNP, with SNPs from PAR1, PAR2, PAR3, and NPR regions colored blue, yellow, red, and black, respectively. (TIFF) [file pgen.1010231.s013.tiff]

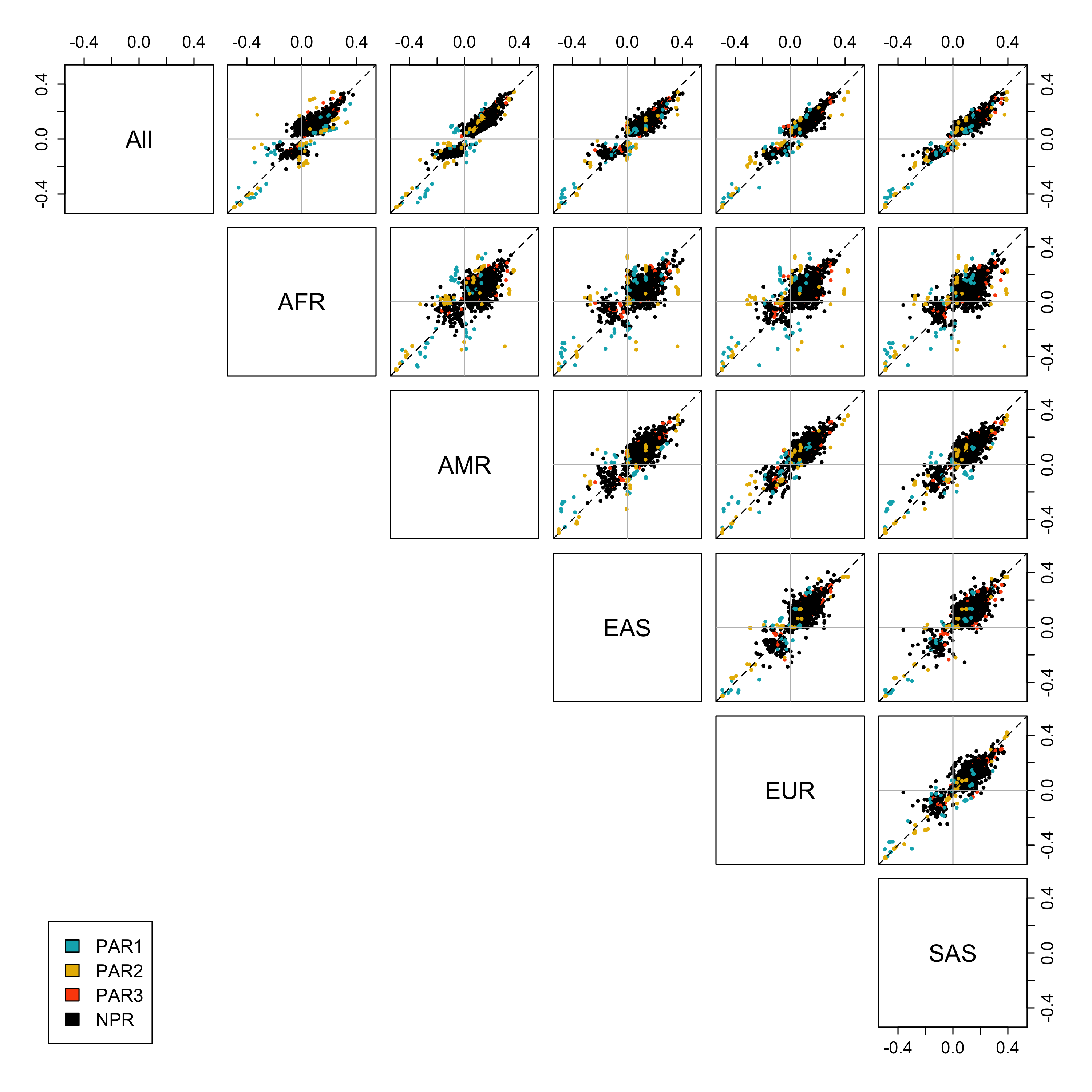

Supplement: S10 Fig — The bi-alleic SNPs shown are the ones with global MAF ≥5% in the combined ALL sample, polymorphic in each of the five superpopulations, and genome-wide significant in at least one of the six sdMAF analyses (i.e. in ALL or any of the five superpopulations). Both X-axis and Y-axis are sdMAF. The dashed line represents locations where X and Y have the same sdMAF. Two solid grey lines represent locations where sdMAF from either group are zeros. Each dot in the scatter plots is colored based on the position of the corresponding SNP, with SNPs from PAR1, PAR2, PAR3 and NPR regions colored blue, yellow, red and black respectively. (TIFF) [file pgen.1010231.s014.tiff]

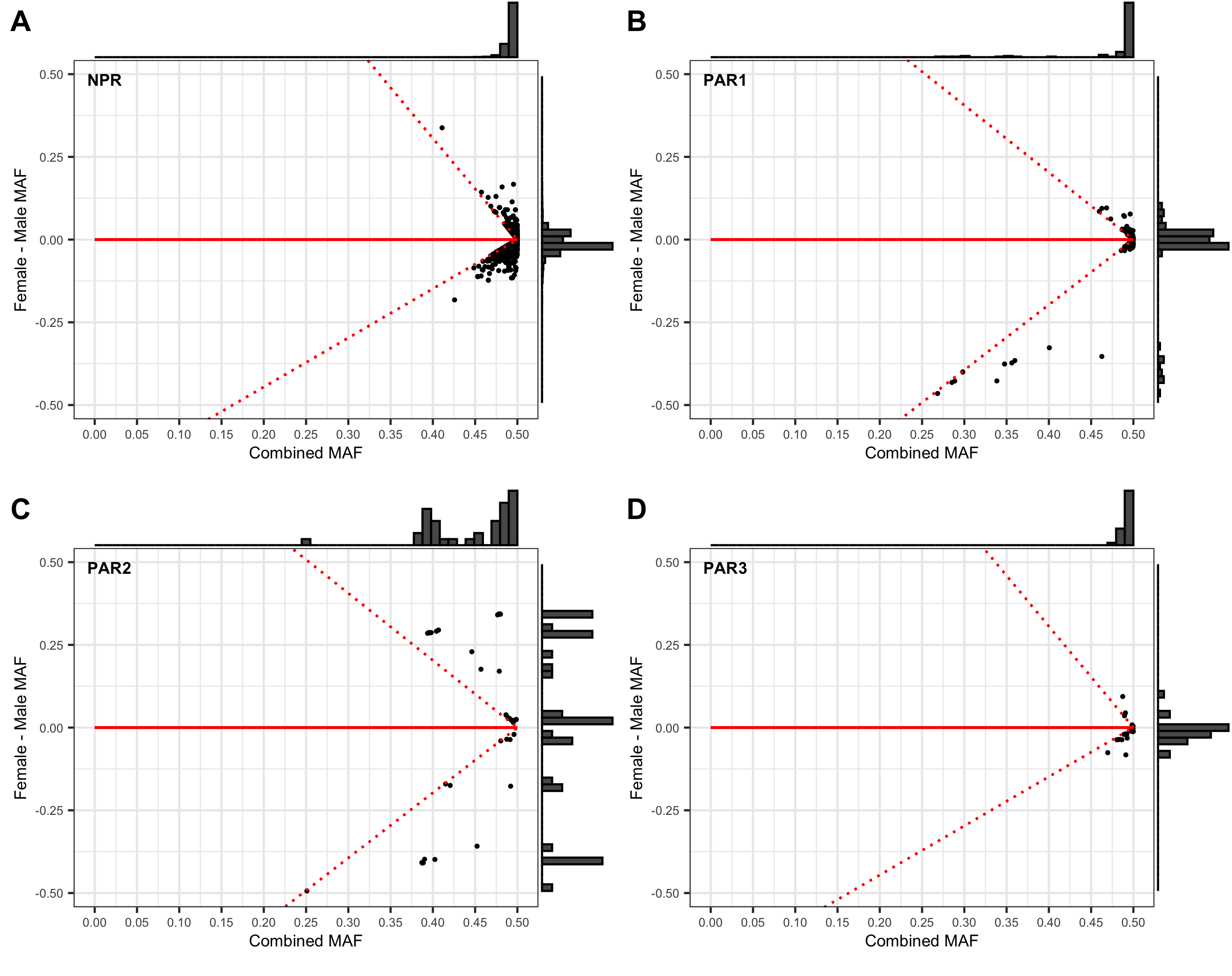

Supplement: S13 Fig — Regions are plotted separately A: NPR; B: PAR1, C: PAR2; D: PAR3. For each of the four regions, the histogram at the top of the Bland-Altman plot shows the distribution of the sex-combined MAF for bi-allelic SNPs with global MAF ≥5% presumed to be of high quality. The histogram to the right of the plot shows the distribution of the Female—Male sdMAF. The red dotted lines are the theoretical bounds; see S3 Note for deviations. (TIFF) [file pgen.1010231.s017.tiff]

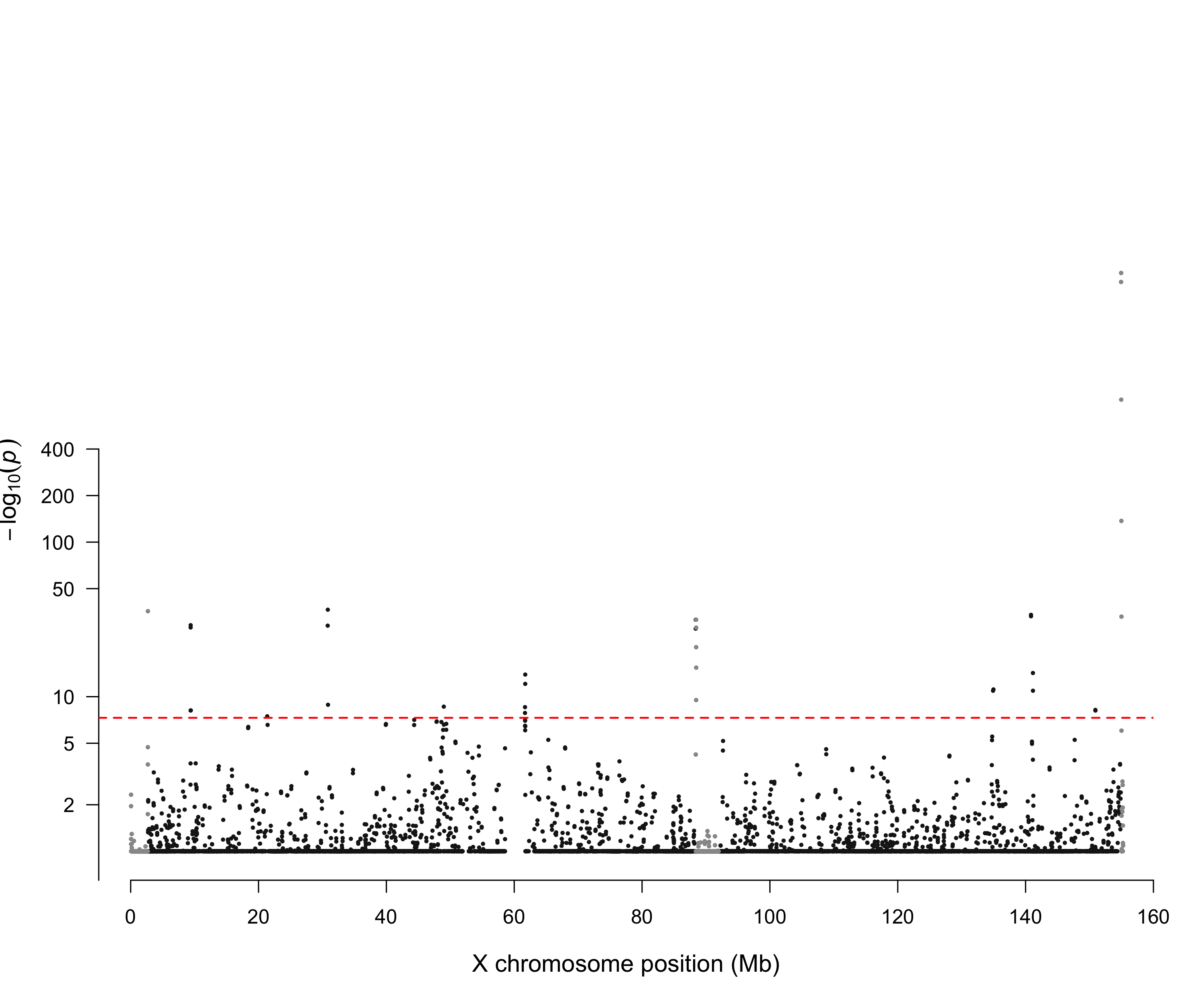

Supplement: S14 Fig — Each window contains 50 adjacent bi-allelic SNPs (with global MAF ≥5%) and are moved by 25 SNPs each time. A sliding window sdMAF p-value (on the–log10 scale) is the average of–log10 p-value of the 50 SNPs in the window. The position of each window is represented by the position of the leftmost SNP. SNPs in the PAR1, PAR2 and PAR3 regions are plotted in grey, with PAR3 located around 90 Mb. Y-axis is -log10(sdMAF p-values) and p-values >0.1 are plotted as 0.1 (1 on -log10 scale) for better visualization. The dashed red line represents 5e-8 (7.3 on the -log10 scale). (TIFF) [file pgen.1010231.s018.tiff]

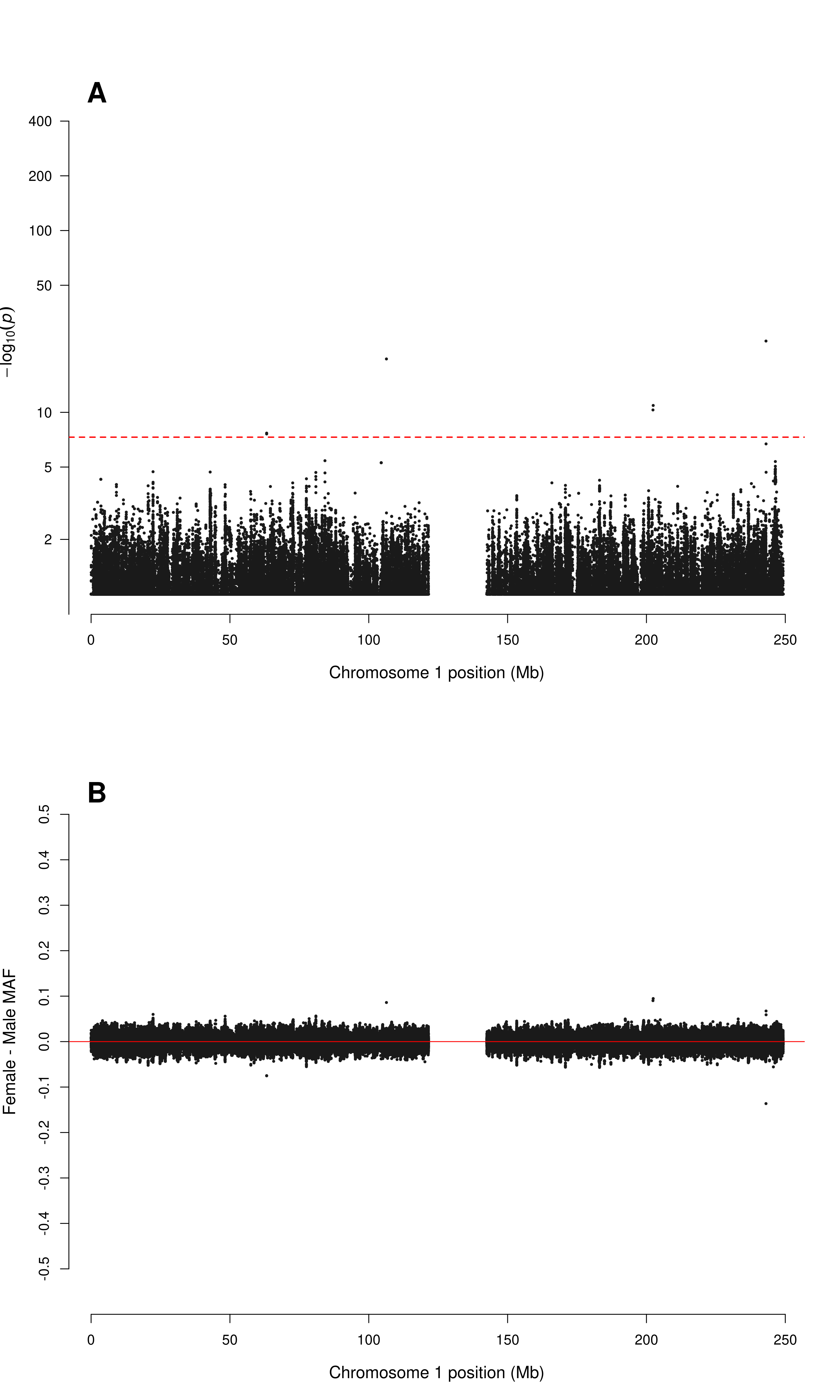

Supplement: S15 Fig — A: sdMAF p-values for bi-allelic SNPs with global MAF ≥5% presumed to be of high quality. Y-axis is −log10(sdMAF p-values) and p-values >0.1 are plotted as 0.1 (1 on −log10 scale) for better visualization. The dashed red line represents 5e-8 (7.3 on the −log10 scale). B: Female—Male sdMAF for the same SNPs in part A. (TIFF) [file pgen.1010231.s019.tiff]

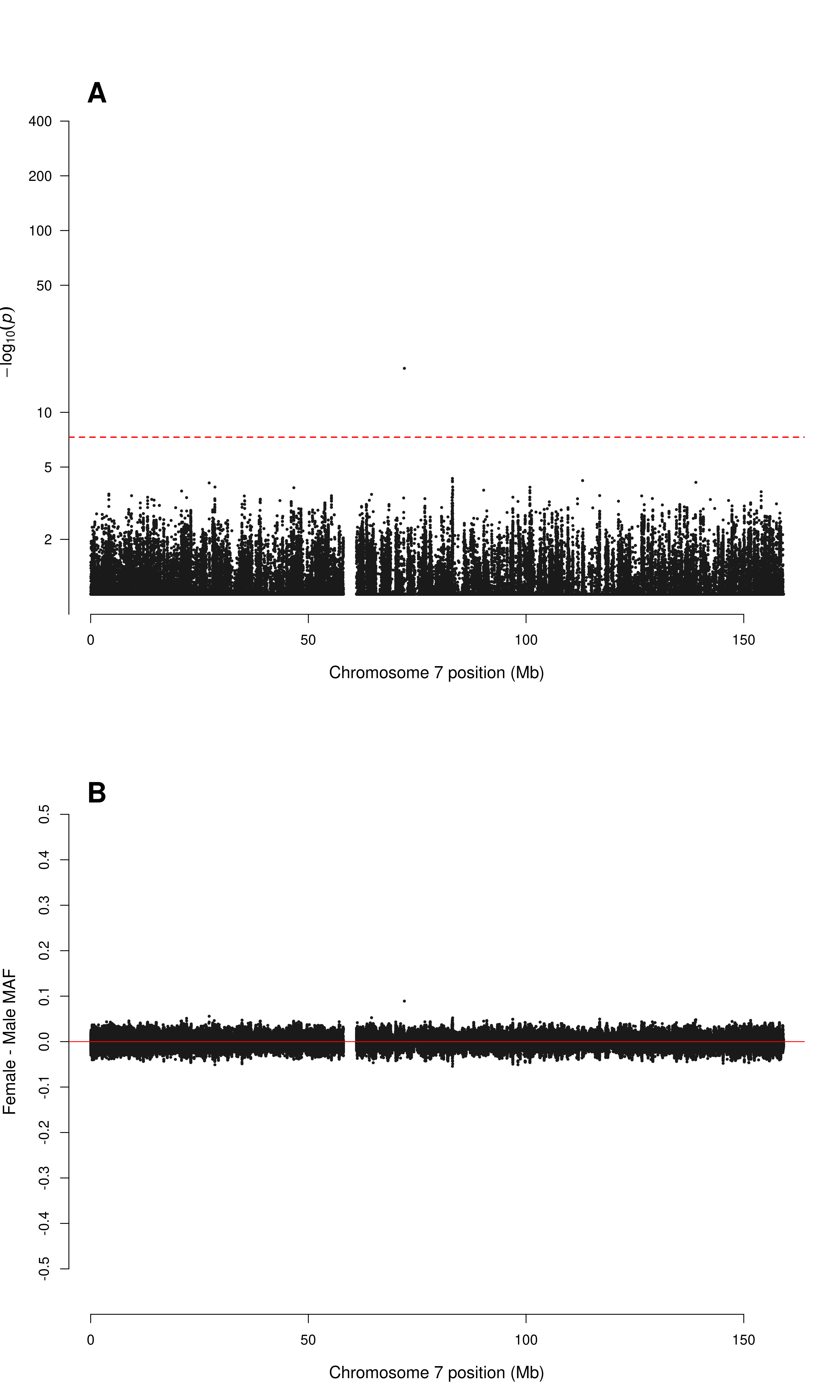

Supplement: S16 Fig — A: sdMAF p-values for bi-allelic SNPs with global MAF ≥5% presumed to be of high quality. Y-axis is −log10(sdMAF p-values) and p-values >0.1 are plotted as 0.1 (1 on −log10 scale) for better visualization. The dashed red line represents 5e-8 (7.3 on the −log10 scale). B: Female—Male sdMAF for the same SNPs in part A. (TIFF) [file pgen.1010231.s020.tiff]

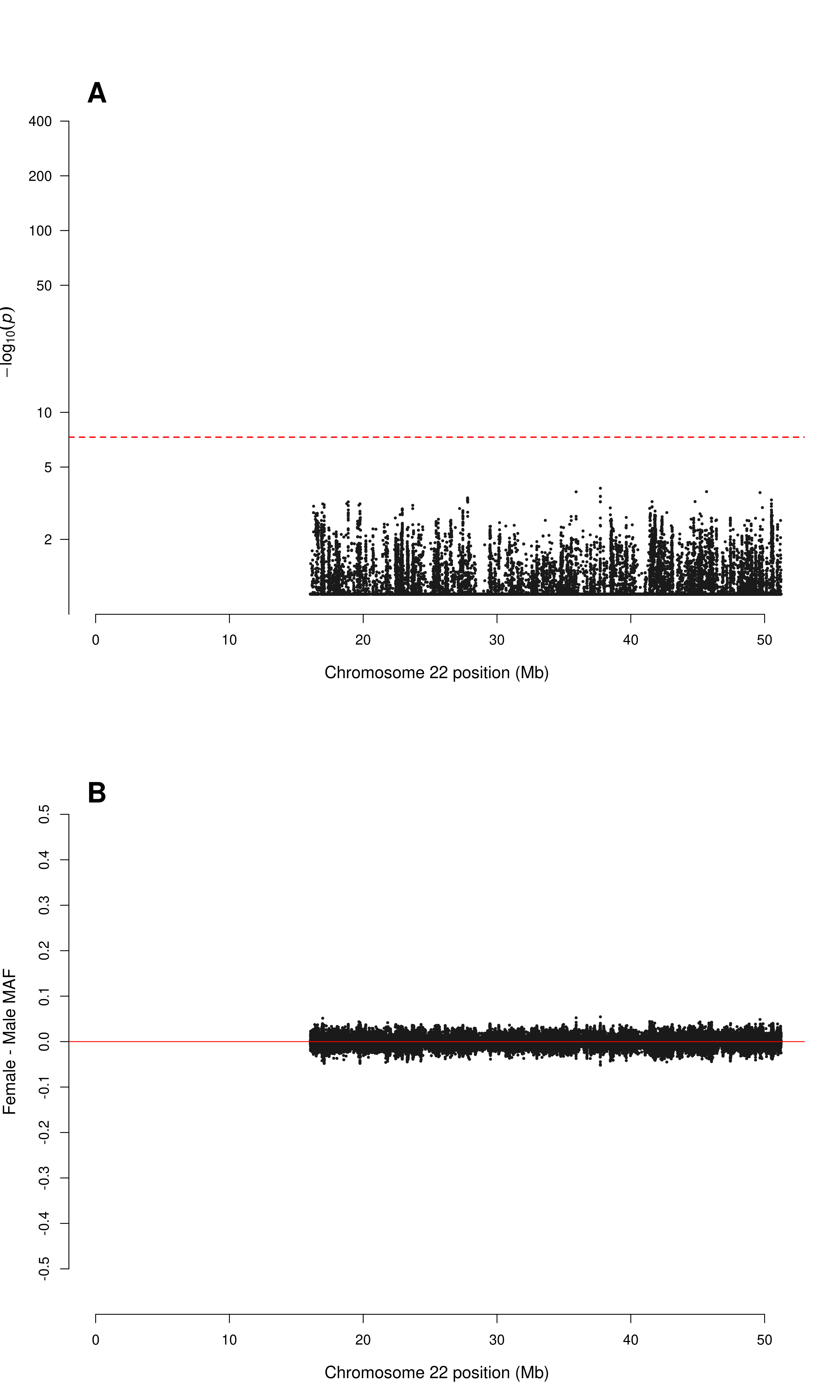

Supplement: S17 Fig — A: sdMAF p-values for bi-allelic SNPs with global MAF ≥5% presumed to be of high quality. Y-axis is −log10(sdMAF p-values) and p-values >0.1 are plotted as 0.1 (1 on −log10 scale) for better visualization. The dashed red line represents 5e-8 (7.3 on the −log10 scale). B: Female—Male sdMAF for the same SNPs in part A. (TIFF) [file pgen.1010231.s021.tiff]

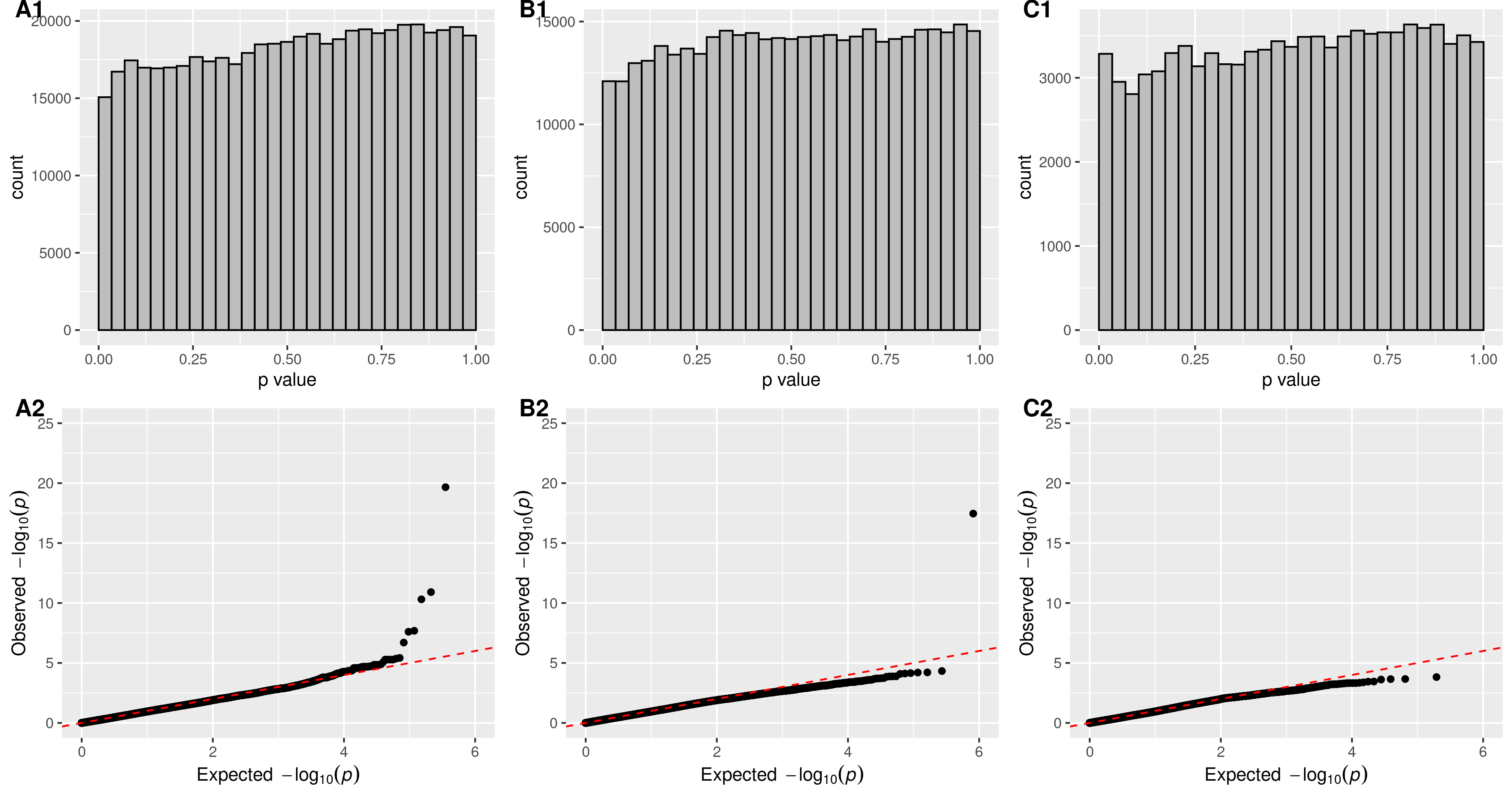

Supplement: S18 Fig — Results of bi-allelic SNPs with global MAF ≥5% are shown separately by chromosome: A; chromosome 1; B: chromosome 7; C: chromosome 22. Unlike the X chromosome results in S2 Fig, there was no truncation of sdMAF p-values at 1e-300 as the smallest sdMAF p-value is around 1e-25 for any of these three autosomes. The red dashed line represents the line of equality. (TIFF) [file pgen.1010231.s022.tiff]

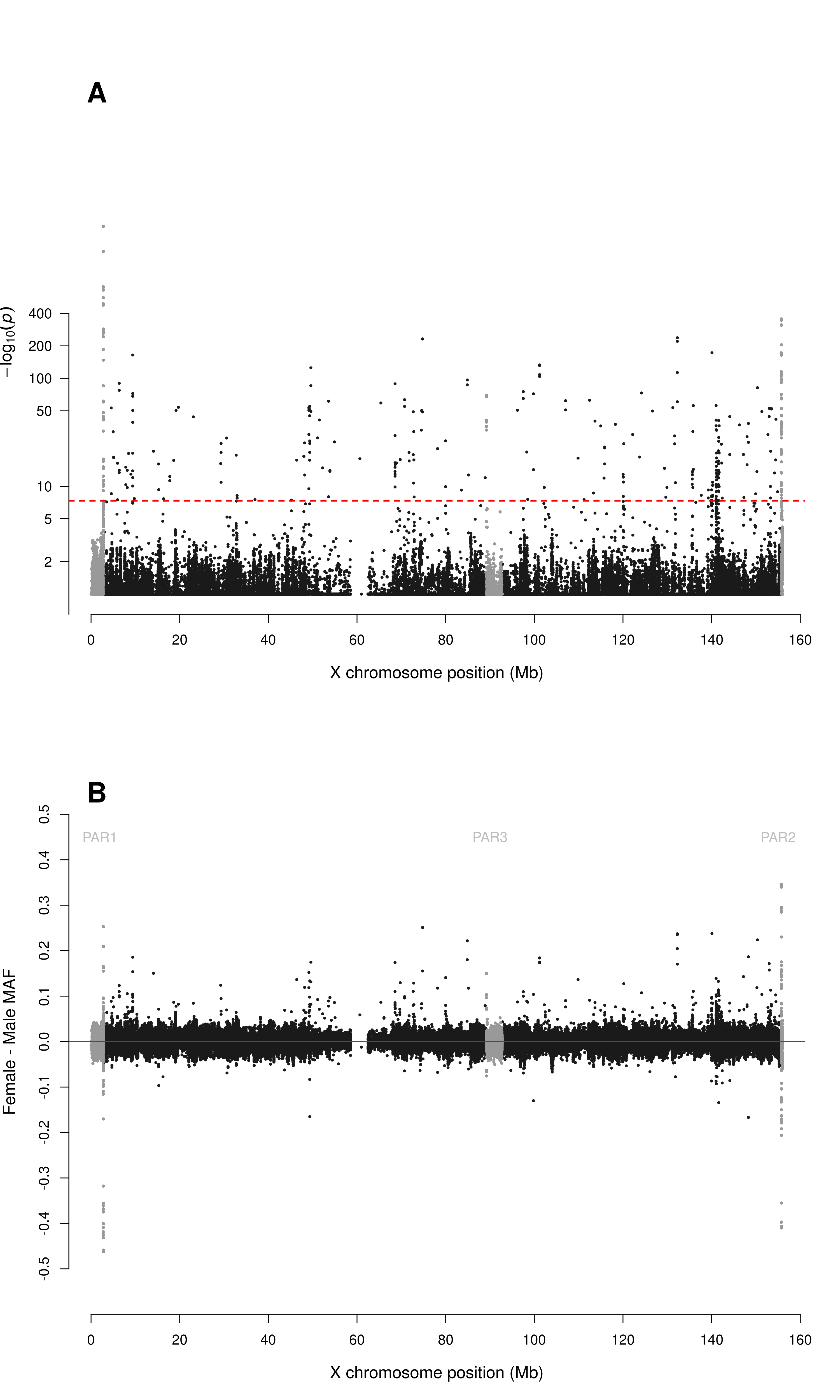

Supplement: S20 Fig — A: sdMAF p-values for bi-allelic SNPs with global MAF ≥5% presumed to be of high quality. SNPs in the PAR1 and PAR3 regions are plotted in grey, with PAR3 located around 90 Mb. Y-axis is −log10(sdMAF p-values) and p-values >0.1 are plotted as 0.1 (1 on −log10 scale) for better visualization. The dashed red line represents 5e-8 (7.3 on the −log10 scale). B: Female—Male sdMAF for the same SNPs in part A. For Zoomed-in plots for the PAR1, PAR2 and PAR3 regions see S21, S22 and S23 Figs, respectively. (TIFF) [file pgen.1010231.s024.tiff]

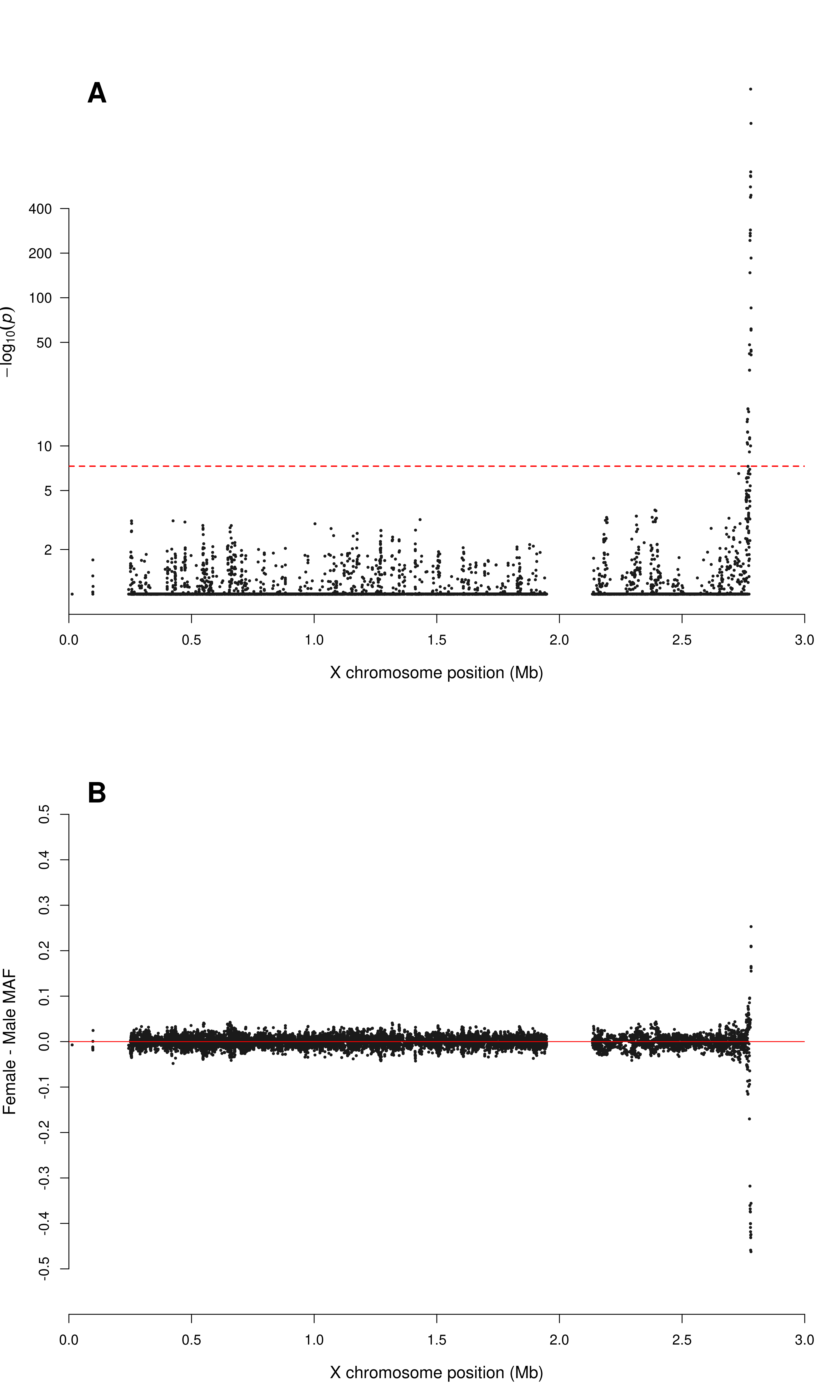

Supplement: S21 Fig — A: sdMAF p-values for bi-allelic SNPs with global MAF ≥5% presumed to be of high quality. Y-axis is −log10(sdMAF p-values) and p-values >0.1 are plotted as 0.1 (1 on −log10 scale) for better visualization. The dashed red line represents 5e-8 (7.3 on the −log10 scale). B: Female—Male sdMAF for the same SNPs in part A. (TIFF) [file pgen.1010231.s025.tiff]

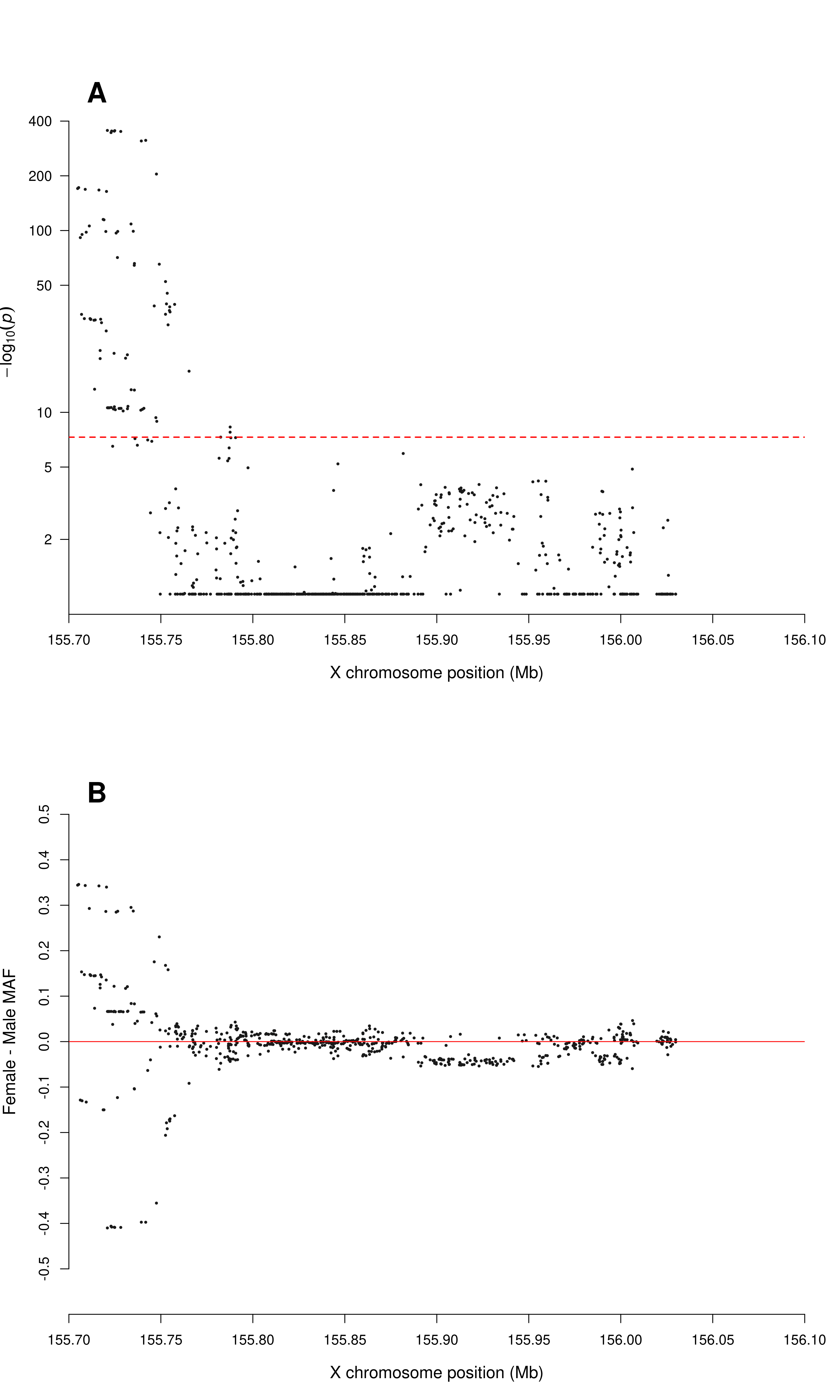

Supplement: S22 Fig — A: sdMAF p-values for bi-allelic SNPs with global MAF ≥5% presumed to be of high quality. Y-axis is −log10(sdMAF p-values) and p-values >0.1 are plotted as 0.1 (1 on −log10 scale) for better visualization. The dashed red line represents 5e-8 (7.3 on the −log10 scale). B: Female—Male sdMAF for the same SNPs in part A. (TIFF) [file pgen.1010231.s026.tiff]

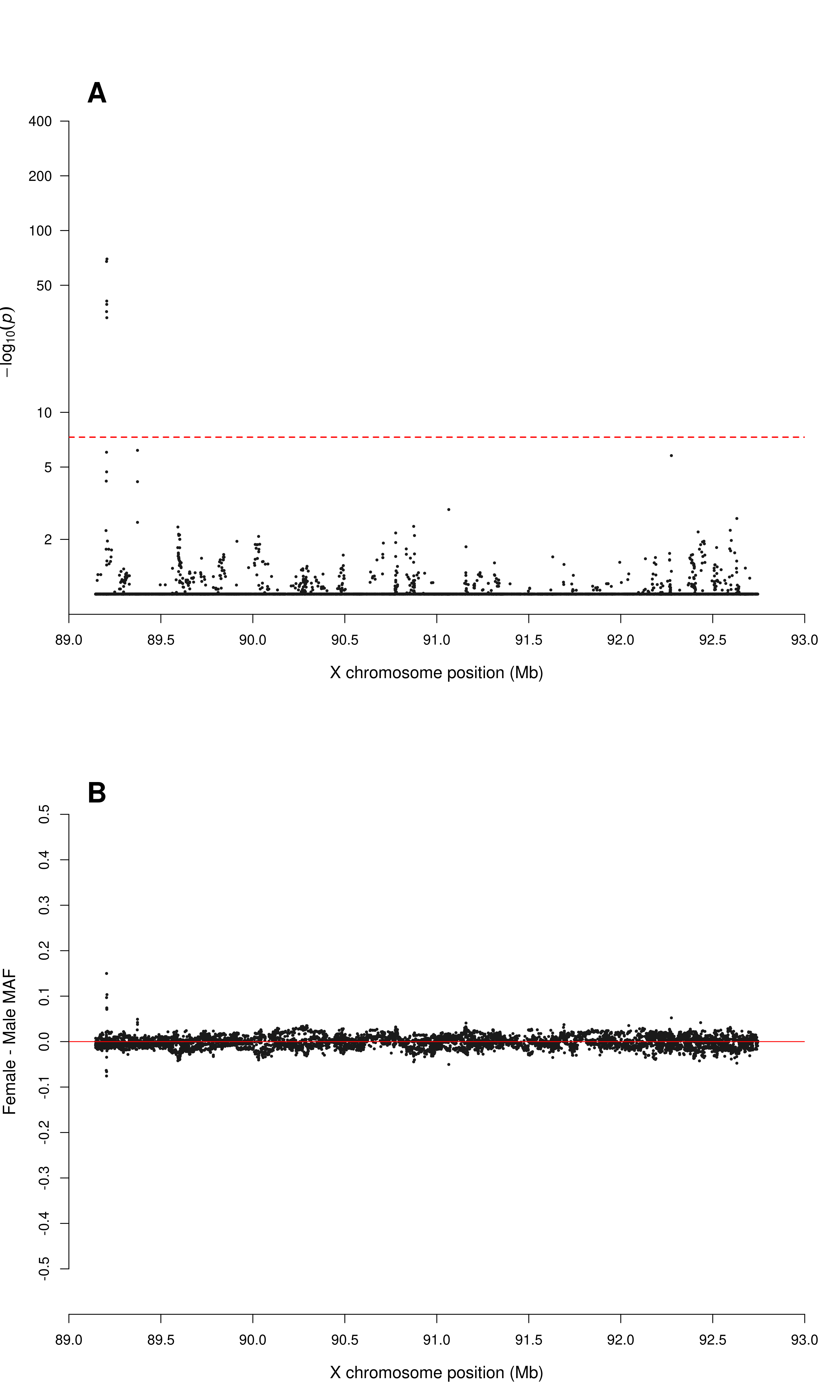

Supplement: S23 Fig — A: sdMAF p-values for bi-allelic SNPs with global MAF ≥5% presumed to be of high quality. Y-axis is −log10(sdMAF p-values) and p-values >0.1 are plotted as 0.1 (1 on −log10 scale) for better visualization. The dashed red line represents 5e-8 (7.3 on the −log10 scale). B: Female—Male sdMAF for the same SNPs in part A. (TIFF) [file pgen.1010231.s027.tiff]

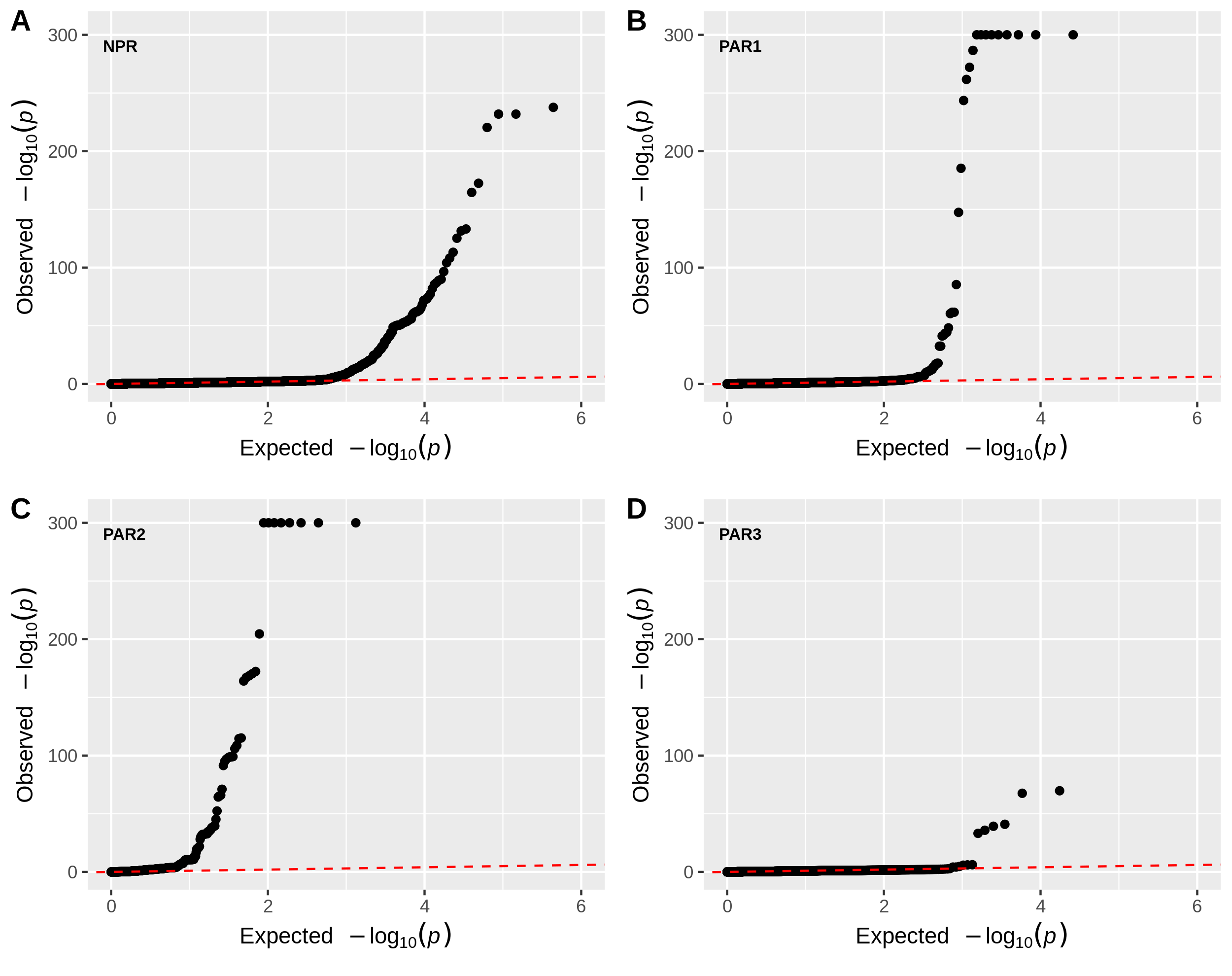

Supplement: S24 Fig — Results of bi-allelic SNPs with global MAF ≥5% are shown separately by region, A: NPR; B: PAR1, C: PAR2; D: PAR3. For better visualization p-values < 1e-300 are plotted as 1e-300 (300 on -log10 scale). The red dashed line represents the line of equality. The corresponding Manhattan plots are in S20 Fig (across the whole X chromosome) and S21, S22 and S23 Figs for PAR1, PAR2 and PAR3, respectively. (TIFF) [file pgen.1010231.s028.tiff]

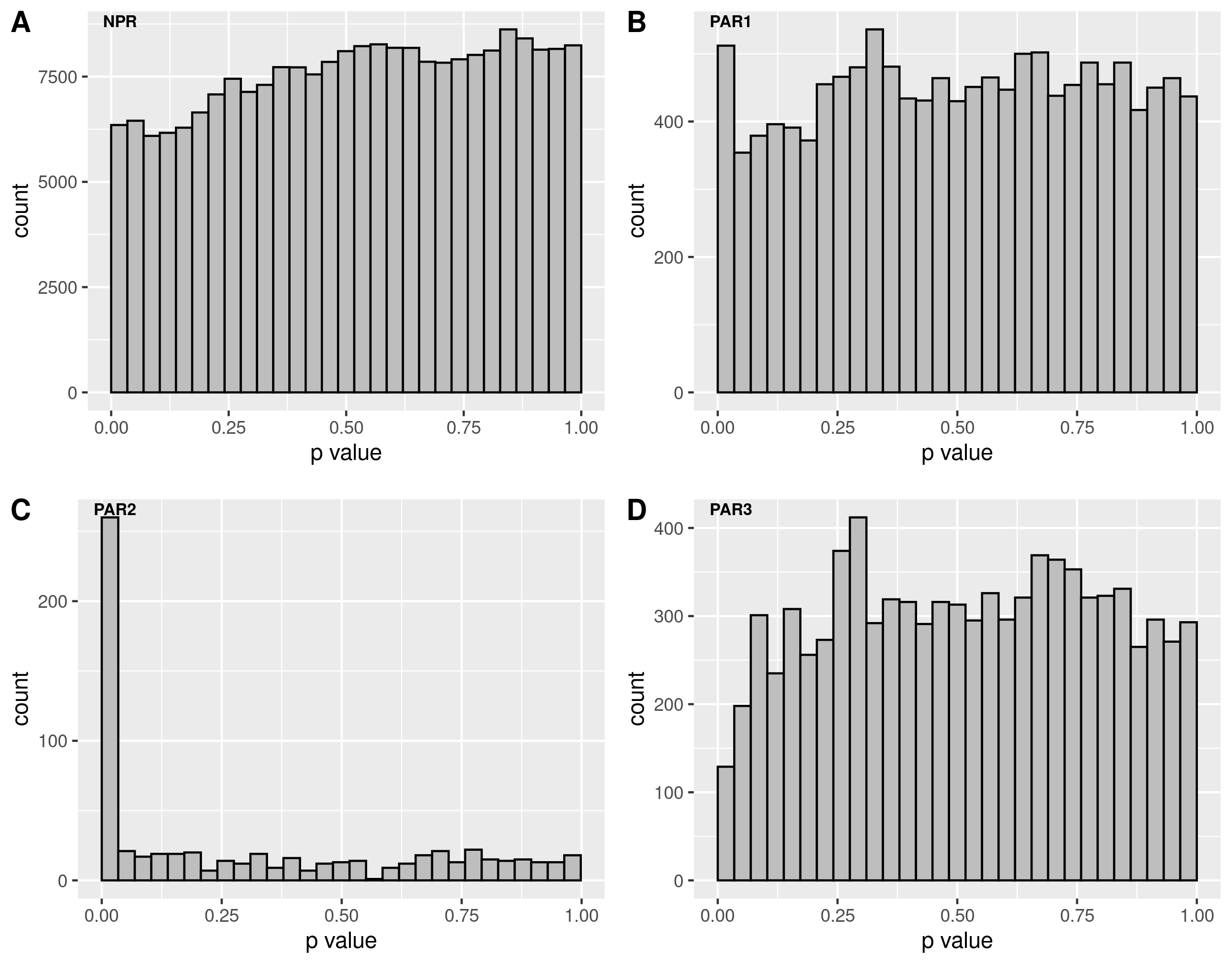

Supplement: S25 Fig — Results of bi-allelic SNPs with global MAF ≥5% are shown separately by region, A: NPR; B: PAR1, C: PAR2; D: PAR3. The corresponding Manhattan plots are in S20 Fig (across the whole X chromosome) and S21, S22 and S23 Figs for PAR1, PAR2 and PAR3, respectively. (TIFF) [file pgen.1010231.s029.tiff]

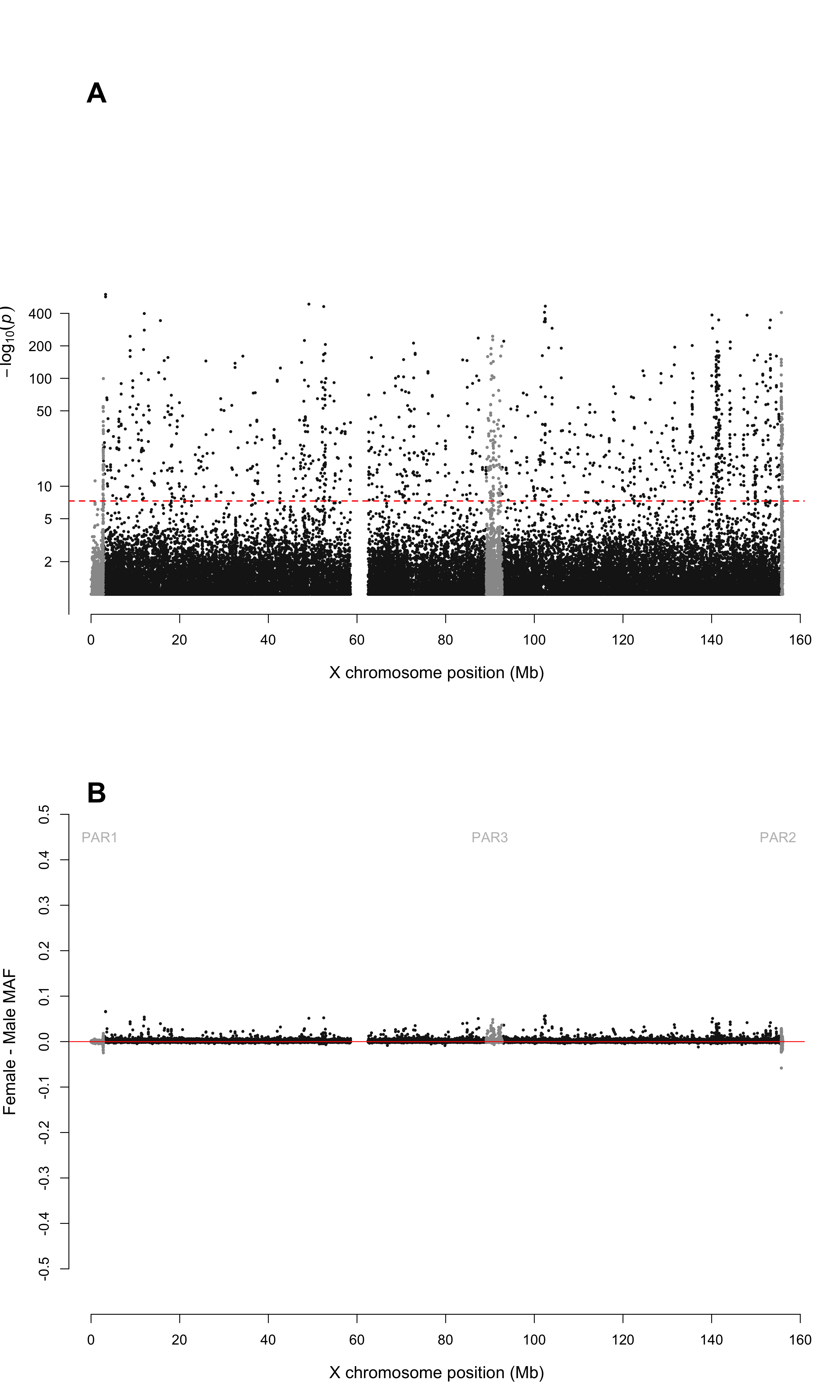

Supplement: S28 Fig — A: sdMAF p-values for bi-allelic SNPs with 0.1%<MAF<5%. SNPs in the PAR1, PAR2 and PAR3 regions are plotted in grey, with PAR3 located around 90 Mb. Y-axis is -log10(sdMAF p-values) and p-values >0.1 are plotted as 0.1 (1 on -log10 scale) for better visualization. The dashed red line represents 5e-8 (7.3 on the -log10 scale). B: Female—Male sdMAF for the same SNPs in part A. (TIFF) [file pgen.1010231.s032.tiff]
