# Supplementary material for: Major sex differences in allele frequencies for X chromosomal variants in both the 1000 Genomes Project and gnomAD
Source: PLoS Genet. 2022 May 31;18(5):e1010231. doi: 10.1371/journal.pgen.1010231 (PMC9187127; doi:10.1371/journal.pgen.1010231)
Supplement: S4 Table — Notations of genotype counts for a biallelic SNP on the X chromosome in (A) the NPR and PAR3 regions and (B) the PAR1 and PAR2 regions. * means not applicable. (PDF) [file pgen.1010231.s004.pdf]

**A**				
Female	aa	Aa	AA	Total
	f_0	f_1	f_2	f
Male	a	*	A	
	m_0	*	m_2	m
Total	n_0	$n_1 (= f_1)$	n_2	n
**B**				
Female	aa	Aa	AA	
	f_0	f_1	f_2	f
Male	aa	Aa	AA	
	m_0	m_1	m_2	m
Total	n_0	n_1	n_2	n
