# Supplementary material for: Major sex differences in allele frequencies for X chromosomal variants in both the 1000 Genomes Project and gnomAD
Source: PLoS Genet. 2022 May 31;18(5):e1010231. doi: 10.1371/journal.pgen.1010231 (PMC9187127; doi:10.1371/journal.pgen.1010231)
Supplement: S19 Fig — Variants were placed into the NPR, PAR1, PAR2, and PAR3 regions based on positions available from The Genome Reference Consortium and (19). For detailed counts of variant types and global MAF by regions, see S3 Table. (PDF) [file pgen.1010231.s023.pdf]

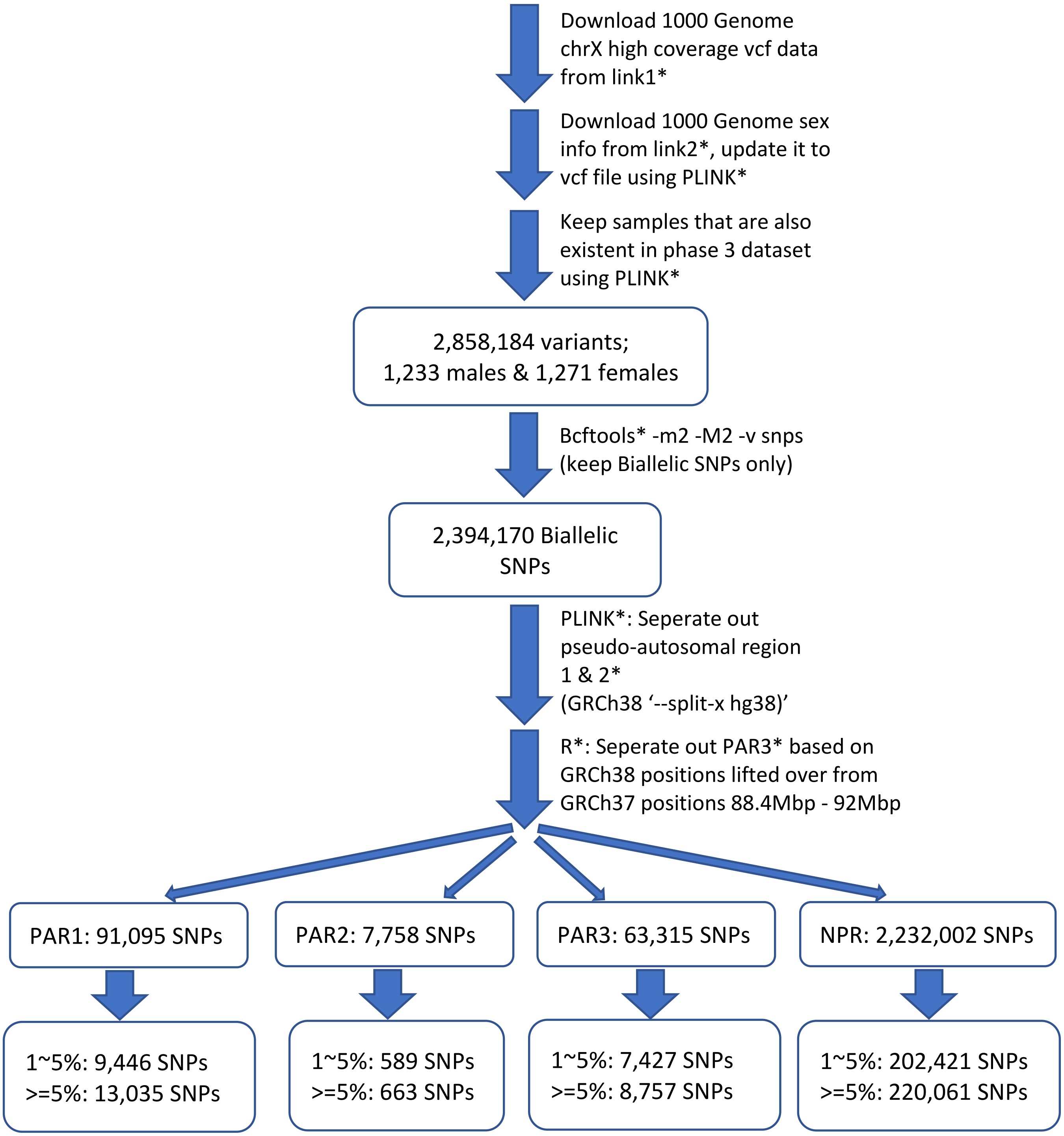

*link1: http://ftp.1000genomes.ebi.ac.uk/vol1/ftp/data_collections/1000G_2504_high_coverage/working/20201028_3202_phased/CCDG_14151_B01_GRM_WGS_2020-08-05_chrX.filtered.eagle2-phased.v2.vcf.gz
*link2: https://www.internationalgenome.org/data-portal/sample
*PLINK: 1.90 beta version 6.20 64-bit
*PAR1&PAR2 exact location
*vcftools: version 0.1.17
*bcftools: version 1.11
*R: version 3.5.3
*PAR3: No global consensus yet
