# Supplementary material for: Major sex differences in allele frequencies for X chromosomal variants in both the 1000 Genomes Project and gnomAD
Source: PLoS Genet. 2022 May 31;18(5):e1010231. doi: 10.1371/journal.pgen.1010231 (PMC9187127; doi:10.1371/journal.pgen.1010231)
Supplement: S27 Fig — Variants were placed into the NPR, PAR1, PAR2, and PAR3 regions based on positions available from The Genome Reference Consortium and (19). (PDF) [file pgen.1010231.s031.pdf]

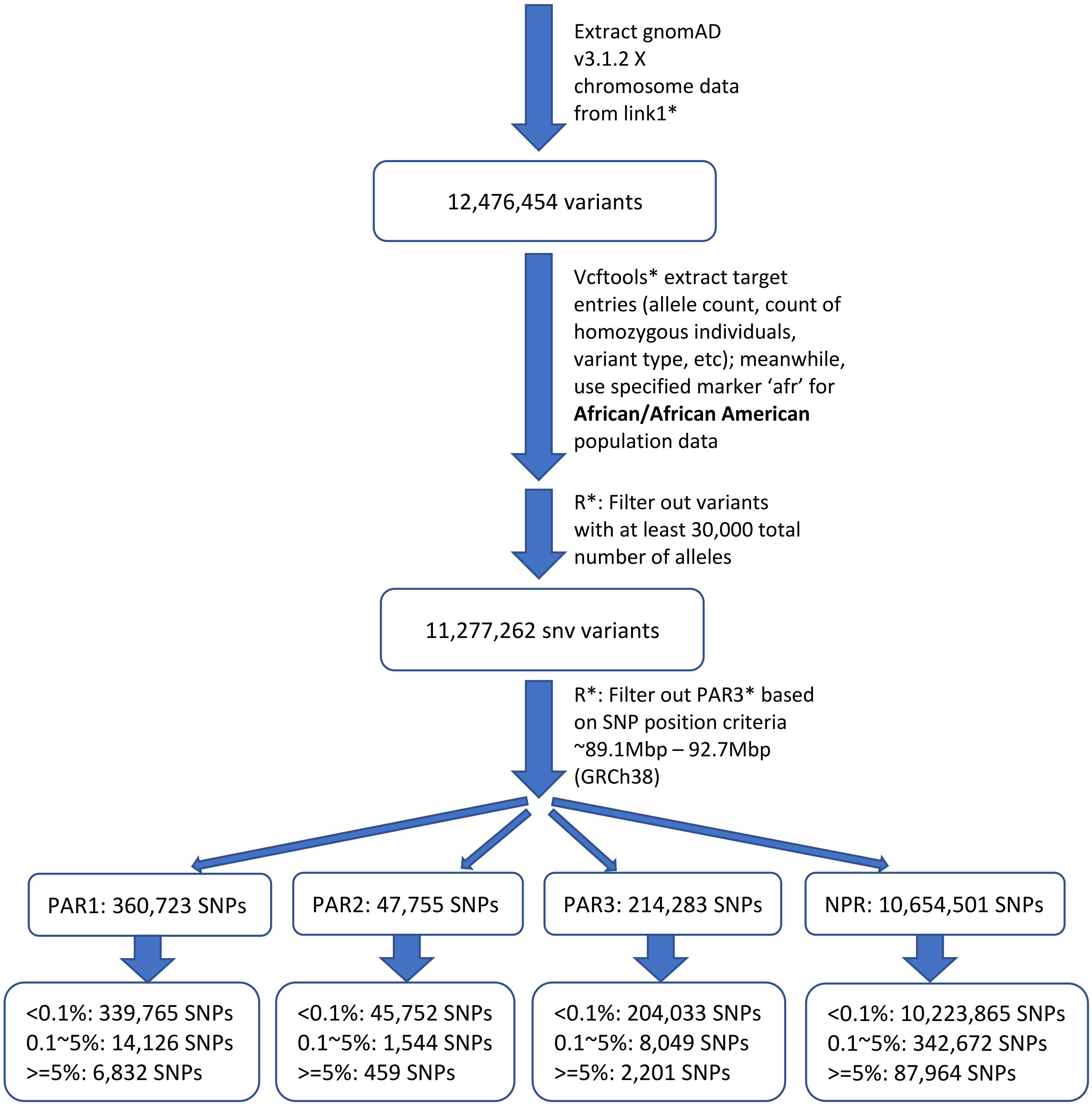

*link1: https://storage.googleapis.com/gcp-public-data--gnomad/release/3.1.2/vcf/genomes/gnomad.genomes.v3.1.2.sites.chrX.vcf.bgz
*PLINK: 1.90 beta version 6.20 64-bit
*vcftools: version 0.1.17
*R: version 3.5.3
*PAR3: No global consensus yet
