# Supplementary material for: Major sex differences in allele frequencies for X chromosomal variants in both the 1000 Genomes Project and gnomAD
Source: PLoS Genet. 2022 May 31;18(5):e1010231. doi: 10.1371/journal.pgen.1010231 (PMC9187127; doi:10.1371/journal.pgen.1010231)
Supplement: S2 Note — In total, 50, 10, 20, and 50 SNPs, respectively from NPR, PAR1, PAR2, and PAR3, with the smallest sdMAF p-values in the phase 3 data were first selected. Among these SNPs, 4, 10, 10, and 9 SNPs, respectively from NPR, PAR1, PAR2, and PAR3, were also bi-allelic in the high coverage data and had no missingness in both sets of data. Each page represents the results for one SNP, and SNPs are ordered by the GRCh37 positions. Within each page, the position of the SNP in phase 3 (build GRCh37) and high coverage (GRCh38) are first provided. Next is the female—male sdMAF difference and the sdMAF p-value. The REF and ALT alleles are also provided for each build. Finally, the counts of the agreement of the genotype calls between the phase 3 and the high coverage data are provided, separately by sex. (PDF) [file pgen.1010231.s034.pdf]

Supplementary Note 2

Comparison of genotypes for selected SNPs between phase 3 and high coverage whole genome sequence data from the 1000 Genomes Project.

In total, 50, 10, 20, and 50 SNPs, respectively from NPR, PAR1, PAR2, and PAR3, with the smallest sdMAF p-values in the phase 3 data were first selected.

Among these SNPs, 4, 10, 10, and 9 SNPs, respectively from NPR, PAR1, PAR2, and PAR3, were also bi-allelic in the high coverage data and had no missingness in both sets of data.

Each page represents the results for one SNP, and SNPs are ordered by the GRCh37 positions.

Within each page, the position of the SNP in phase 3 (build GRCh37) and high coverage (GRCh38) are first provided. Next is the female - male sdMAF difference and the sdMAF p-value. The REF and ALT alleles are also provided for each build. Finally, the counts of the agreement of the genotype calls between the phase 3 and the high coverage data are provided, separately by sex.

Region: PAR1
rs number (GRCh37 phase3): .
SNP position (GRCh37) = 2695860 ; Female-Male Sex Difference in MAF (GRCh37 phase3) = -0.3727 ; sdMAF P-value = 8.20918418869952e-268
SNP position (GRCh38) = 2777819 ; Female-Male Sex Difference in MAF (High Coverage) = -0.3739 ; sdMAF P-value = 7.863953925689e-273

Table A: REF/ALT

	REF	ALT
Phase3 (GRCh37)	A	G
High Coverage	A	G

Table B: male

	aa	Aa	AA	Total_P3
aa	99	3	0	102
Aa	0	917	0	917
AA	0	1	213	214
Total_HC	99	921	213	1233

Table C: female

	aa	Aa	AA	Total_P3
aa	892	1	0	893
Aa	0	317	0	317
AA	0	2	59	61
Total_HC	892	320	59	1271

Note1. Row (Phase 3 GRCh37); Column (High Coverage GRCh38)
Note2. ‘a’: REF; ‘A’: ALT

Region: PAR1
rs number (GRCh37 phase3): .
SNP position (GRCh37) = 2696560 ; Female-Male Sex Difference in MAF (GRCh37 phase3) = -0.376 ; sdMAF P-value = 5.54370357180522e-289
SNP position (GRCh38) = 2778519 ; Female-Male Sex Difference in MAF (High Coverage) = -0.3748 ; sdMAF P-value = 2.65832322082409e-287

Table D: REF/ALT

	REF	ALT
Phase3 (GRCh37)	G	A
High Coverage	G	A

Table E: male

	aa	Aa	AA	Total_P3
aa	98	1	0	99
Aa	0	940	0	940
AA	0	2	192	194
Total_HC	98	943	192	1233

Table F: female

	aa	Aa	AA	Total_P3
aa	912	1	0	913
Aa	0	302	1	303
AA	0	0	55	55
Total_HC	912	303	56	1271

Note1. Row (Phase 3 GRCh37); Column (High Coverage GRCh38)
Note2. ‘a’: REF; ‘A’: ALT

Region: PAR1
rs number (GRCh37 phase3): .
SNP position (GRCh37) = 2696892 ; Female-Male Sex Difference in MAF (GRCh37 phase3) = -0.4001 ; sdMAF P-value < 1e-300
SNP position (GRCh38) = 2778851 ; Female-Male Sex Difference in MAF (High Coverage) = -0.40047 ; sdMAF P-value < 1e-300

Table G: REF/ALT

	REF	ALT
Phase3 (GRCh37)	G	A
High Coverage	G	A

Table H: male

	aa	Aa	AA	Total_P3
aa	103	2	0	105
Aa	1	1019	0	1020
AA	0	4	104	108
Total_HC	104	1025	104	1233

Table I: female

	aa	Aa	AA	Total_P3
aa	1041	0	0	1041
Aa	2	201	0	203
AA	0	2	25	27
Total_HC	1043	203	25	1271

Note1. Row (Phase 3 GRCh37); Column (High Coverage GRCh38)
Note2. ‘a’: REF; ‘A’: ALT

Region: PAR1
rs number (GRCh37 phase3): .
SNP position (GRCh37) = 2696893 ; Female-Male Sex Difference in MAF (GRCh37 phase3) = -0.4001 ; sdMAF P-value < 1e-300
SNP position (GRCh38) = 2778852 ; Female-Male Sex Difference in MAF (High Coverage) = -0.40047 ; sdMAF P-value < 1e-300

Table J: REF/ALT

	REF	ALT
Phase3 (GRCh37)	C	T
High Coverage	C	T

Table K: male

	aa	Aa	AA	Total_P3
aa	103	2	0	105
Aa	1	1019	0	1020
AA	0	4	104	108
Total_HC	104	1025	104	1233

Table L: female

	aa	Aa	AA	Total_P3
aa	1041	0	0	1041
Aa	2	201	0	203
AA	0	2	25	27
Total_HC	1043	203	25	1271

Note1. Row (Phase 3 GRCh37); Column (High Coverage GRCh38)
Note2. ‘a’: REF; ‘A’: ALT

Region: PAR1
rs number (GRCh37 phase3): .
SNP position (GRCh37) = 2697154 ; Female-Male Sex Difference in MAF (GRCh37 phase3) = -0.41948 ; sdMAF P-value < 1e-300
SNP position (GRCh38) = 2779113 ; Female-Male Sex Difference in MAF (High Coverage) = -0.4183 ; sdMAF P-value < 1e-300

Table M: REF/ALT

	REF	ALT
Phase3 (GRCh37)	A	C
High Coverage	A	C

Table N: male

	aa	Aa	AA	Total_P3
aa	106	2	0	108
Aa	1	1040	2	1043
AA	0	3	79	82
Total_HC	107	1045	81	1233

Table O: female

	aa	Aa	AA	Total_P3
aa	1097	3	0	1100
Aa	1	162	1	164
AA	0	0	7	7
Total_HC	1098	165	8	1271

Note1. Row (Phase 3 GRCh37); Column (High Coverage GRCh38)
Note2. ‘a’: REF; ‘A’: ALT

Region: PAR1
rs number (GRCh37 phase3): .
SNP position (GRCh37) = 2697599 ; Female-Male Sex Difference in MAF (GRCh37 phase3) = -0.461312 ; sdMAF P-value < 1e-300
SNP position (GRCh38) = 2779558 ; Female-Male Sex Difference in MAF (High Coverage) = -0.458793 ; sdMAF P-value < 1e-300

Table P: REF/ALT

	REF	ALT
Phase3 (GRCh37)	C	A
High Coverage	C	A

Table Q: male

	aa	Aa	AA	Total_P3
aa	88	2	0	90
Aa	7	1125	0	1132
AA	0	4	7	11
Total_HC	95	1131	7	1233

Table R: female

	aa	Aa	AA	Total_P3
aa	1253	1	0	1254
Aa	4	13	0	17
AA	0	0	0	0
Total_HC	1257	14	0	1271

Note1. Row (Phase 3 GRCh37); Column (High Coverage GRCh38)
Note2. ‘a’: REF; ‘A’: ALT

Region: PAR1
rs number (GRCh37 phase3): .
SNP position (GRCh37) = 2697845 ; Female-Male Sex Difference in MAF (GRCh37 phase3) = -0.42742 ; sdMAF P-value < 1e-300
SNP position (GRCh38) = 2779804 ; Female-Male Sex Difference in MAF (High Coverage) = -0.42582 ; sdMAF P-value < 1e-300

Table S: REF/ALT

	REF	ALT
Phase3 (GRCh37)	C	T
High Coverage	C	T

Table T: male

	aa	Aa	AA	Total_P3
aa	78	2	0	80
Aa	1	1057	1	1059
AA	0	5	89	94
Total_HC	79	1064	90	1233

Table U: female

	aa	Aa	AA	Total_P3
aa	1089	4	0	1093
Aa	2	155	0	157
AA	0	1	20	21
Total_HC	1091	160	20	1271

Note1. Row (Phase 3 GRCh37); Column (High Coverage GRCh38)
Note2. ‘a’: REF; ‘A’: ALT

Region: PAR1
rs number (GRCh37 phase3): .
SNP position (GRCh37) = 2697868 ; Female-Male Sex Difference in MAF (GRCh37 phase3) = -0.43172 ; sdMAF P-value < 1e-300
SNP position (GRCh38) = 2779827 ; Female-Male Sex Difference in MAF (High Coverage) = -0.43133 ; sdMAF P-value < 1e-300

Table V: REF/ALT

	REF	ALT
Phase3 (GRCh37)	G	A
High Coverage	G	A

Table W: male

	aa	Aa	AA	Total_P3
aa	78	2	0	80
Aa	0	1059	3	1062
AA	0	5	86	91
Total_HC	78	1066	89	1233

Table X: female

	aa	Aa	AA	Total_P3
aa	1100	4	0	1104
Aa	2	147	0	149
AA	0	1	17	18
Total_HC	1102	152	17	1271

Note1. Row (Phase 3 GRCh37); Column (High Coverage GRCh38)
Note2. ‘a’: REF; ‘A’: ALT

Region: PAR1
rs number (GRCh37 phase3): .
SNP position (GRCh37) = 2698923 ; Female-Male Sex Difference in MAF (GRCh37 phase3) = -0.46516 ; sdMAF P-value < 1e-300
SNP position (GRCh38) = 2780882 ; Female-Male Sex Difference in MAF (High Coverage) = -0.46225 ; sdMAF P-value < 1e-300

Table Y: REF/ALT

	REF	ALT
Phase3 (GRCh37)	G	A
High Coverage	G	A

Table Z: male

	aa	Aa	AA	Total_P3
aa	38	6	0	44
Aa	6	1128	0	1134
AA	0	8	47	55
Total_HC	44	1142	47	1233

Table AA: female

	aa	Aa	AA	Total_P3
aa	1178	0	0	1178
Aa	0	85	1	86
AA	0	2	5	7
Total_HC	1178	87	6	1271

Note1. Row (Phase 3 GRCh37); Column (High Coverage GRCh38)
Note2. ‘a’: REF; ‘A’: ALT

Region: PAR1
rs number (GRCh37 phase3): .
SNP position (GRCh37) = 2698954 ; Female-Male Sex Difference in MAF (GRCh37 phase3) = -0.427 ; sdMAF P-value < 1e-300
SNP position (GRCh38) = 2780913 ; Female-Male Sex Difference in MAF (High Coverage) = -0.4245 ; sdMAF P-value < 1e-300

Table AB: REF/ALT

	REF	ALT
Phase3 (GRCh37)	G	A
High Coverage	G	A

Table AC: male

	aa	Aa	AA	Total_P3
aa	37	5	0	42
Aa	1	1011	1	1013
AA	0	10	168	178
Total_HC	38	1026	169	1233

Table AD: female

	aa	Aa	AA	Total_P3
aa	975	4	0	979
Aa	3	254	1	258
AA	0	1	33	34
Total_HC	978	259	34	1271

Note1. Row (Phase 3 GRCh37); Column (High Coverage GRCh38)
Note2. 'a': REF; 'A': ALT

Region: NPR
rs number (GRCh37 phase3): rs1996225
SNP position (GRCh37) = 15711209 ; Female-Male Sex Difference in MAF (GRCh37 phase3) = 0.2344 ; sdMAF P-value = 1.55380206985061e-60
SNP position (GRCh38) = 15693086 ; Female-Male Sex Difference in MAF (High Coverage) = 0.0352 ; sdMAF P-value = 0.0251232744816044

Table AE: REF/ALT

	REF	ALT
Phase3 (GRCh37)	C	T
High Coverage	C	T

Table AF: male

	a	A	Total_P3
a	315	11	326
A	0	907	907
Total_HC	315	918	1233

Table AG: female

	aa	Aa	AA	Total_P3
aa	147	1	0	148
Aa	0	444	528	972
AA	0	0	151	151
Total_HC	147	445	679	1271

Note1. Row (Phase 3 GRCh37); Column (High Coverage GRCh38)
Note2. ‘a’: REF; ‘A’: ALT

Region: NPR
rs number (GRCh37 phase3): rs372984882
SNP position (GRCh37) = 88383111 ; Female-Male Sex Difference in MAF (GRCh37 phase3) = 0.28941 ; sdMAF P-value = 3.9737450506146e-213
SNP position (GRCh38) = 89128111 ; Female-Male Sex Difference in MAF (High Coverage) = 0.00236 ; sdMAF P-value = 0.0140770215674526

Table AH: REF/ALT

	REF	ALT
Phase3 (GRCh37)	A	T
High Coverage	A	T

Table AI: male

	a	A	Total_P3
a	1161	0	1161
A	72	0	72
Total_HC	1233	0	1233

Table AJ: female

	aa	Aa	AA	Total_P3
aa	384	3	0	387
Aa	881	3	0	884
AA	0	0	0	0
Total_HC	1265	6	0	1271

Note1. Row (Phase 3 GRCh37); Column (High Coverage GRCh38)
Note2. ‘a’: REF; ‘A’: ALT

Region: NPR
rs number (GRCh37 phase3): rs369028615
SNP position (GRCh37) = 88383115 ; Female-Male Sex Difference in MAF (GRCh37 phase3) = 0.28941 ; sdMAF P-value = 3.9737450506146e-213
SNP position (GRCh38) = 89128115 ; Female-Male Sex Difference in MAF (High Coverage) = 0.00236 ; sdMAF P-value = 0.0140770215674526

Table AK: REF/ALT

	REF	ALT
Phase3 (GRCh37)	C	T
High Coverage	C	T

Table AL: male

	a	A	Total_P3
a	1161	0	1161
A	72	0	72
Total_HC	1233	0	1233

Table AM: female

	aa	Aa	AA	Total_P3
aa	384	3	0	387
Aa	881	3	0	884
AA	0	0	0	0
Total_HC	1265	6	0	1271

Note1. Row (Phase 3 GRCh37); Column (High Coverage GRCh38)
Note2. ‘a’: REF; ‘A’: ALT

Region: PAR3
rs number (GRCh37 phase3): .
SNP position (GRCh37) = 88458816 ; Female-Male Sex Difference in MAF (GRCh37 phase3) = 0.16151 ; sdMAF P-value = 2.25662814279251e-56
SNP position (GRCh38) = 89203817 ; Female-Male Sex Difference in MAF (High Coverage) = 0.04013 ; sdMAF P-value = 6.22346860302191e-26

Table AN: REF/ALT

	REF	ALT
Phase3 (GRCh37)	G	A
High Coverage	G	A

Table AO: male

	a	A	Total_P3
a	1143	0	1143
A	90	0	90
Total_HC	1233	0	1233

Table AP: female

	aa	Aa	AA	Total_P3
aa	649	27	0	676
Aa	519	75	0	594
AA	1	0	0	1
Total_HC	1169	102	0	1271

Note1. Row (Phase 3 GRCh37); Column (High Coverage GRCh38)
Note2. ‘a’: REF; ‘A’: ALT

Region: PAR3
rs number (GRCh37 phase3): .
SNP position (GRCh37) = 88458918 ; Female-Male Sex Difference in MAF (GRCh37 phase3) = 0.1504 ; sdMAF P-value = 3.10020205638595e-48
SNP position (GRCh38) = 89203919 ; Female-Male Sex Difference in MAF (High Coverage) = 0.04917 ; sdMAF P-value = 5.29484546944359e-32

Table AQ: REF/ALT

	REF	ALT
Phase3 (GRCh37)	A	G
High Coverage	A	G

Table AR: male

	a	A	Total_P3
a	1142	0	1142
A	91	0	91
Total_HC	1233	0	1233

Table AS: female

	aa	Aa	AA	Total_P3
aa	651	57	0	708
Aa	490	66	0	556
AA	5	2	0	7
Total_HC	1146	125	0	1271

Note1. Row (Phase 3 GRCh37); Column (High Coverage GRCh38)
Note2. ‘a’: REF; ‘A’: ALT

Region: PAR3
rs number (GRCh37 phase3): rs370154167
SNP position (GRCh37) = 88460271 ; Female-Male Sex Difference in MAF (GRCh37 phase3) = 0.2629 ; sdMAF P-value = 9.87188301068426e-132
SNP position (GRCh38) = 89205272 ; Female-Male Sex Difference in MAF (High Coverage) = 0.002754 ; sdMAF P-value = 0.00797639777080226

Table AT: REF/ALT

	REF	ALT
Phase3 (GRCh37)	G	A
High Coverage	G	A

Table AU: male

	a	A	Total_P3
a	1108	0	1108
A	125	0	125
Total_HC	1233	0	1233

Table AV: female

	aa	Aa	AA	Total_P3
aa	354	1	0	355
Aa	900	6	0	906
AA	10	0	0	10
Total_HC	1264	7	0	1271

Note1. Row (Phase 3 GRCh37); Column (High Coverage GRCh38)
Note2. 'a': REF; 'A': ALT

Region: PAR3
rs number (GRCh37 phase3): .
SNP position (GRCh37) = 88460457 ; Female-Male Sex Difference in MAF (GRCh37 phase3) = 0.125 ; sdMAF P-value = 3.85096879960068e-45
SNP position (GRCh38) = 89205458 ; Female-Male Sex Difference in MAF (High Coverage) = -2.42e-05 ; sdMAF P-value = 0.980320072234104

Table AW: REF/ALT

	REF	ALT
Phase3 (GRCh37)	G	A
High Coverage	G	C

Table AX: male

	a	A	Total_P3
a	1178	1	1179
A	54	0	54
Total_HC	1232	1	1233

Table AY: female

	aa	Aa	AA	Total_P3
aa	842	2	0	844
Aa	425	0	0	425
AA	2	0	0	2
Total_HC	1269	2	0	1271

Note1. Row (Phase 3 GRCh37); Column (High Coverage GRCh38)
Note2. ‘a’: REF; ‘A’: ALT

Region: PAR3
rs number (GRCh37 phase3): .
SNP position (GRCh37) = 88460883 ; Female-Male Sex Difference in MAF (GRCh37 phase3) = 0.0985 ; sdMAF P-value = 3.54405130280993e-13
SNP position (GRCh38) = 89205884 ; Female-Male Sex Difference in MAF (High Coverage) = 0.015911 ; sdMAF P-value = 3.74164715957335e-05

Table AZ: REF/ALT

	REF	ALT
Phase3 (GRCh37)	G	A
High Coverage	G	A

Table BA: male

	a	A	Total_P3
a	978	5	983
A	246	4	250
Total_HC	1224	9	1233

Table BB: female

	aa	Aa	AA	Total_P3
aa	504	19	0	523
Aa	692	37	1	730
AA	17	1	0	18
Total_HC	1213	57	1	1271

Note1. Row (Phase 3 GRCh37); Column (High Coverage GRCh38)
Note2. ‘a’: REF; ‘A’: ALT

Region: PAR3
rs number (GRCh37 phase3): rs199820323
SNP position (GRCh37) = 89192798 ; Female-Male Sex Difference in MAF (GRCh37 phase3) = 0.10024 ; sdMAF P-value = 4.78641113314677e-32
SNP position (GRCh38) = 89937799 ; Female-Male Sex Difference in MAF (High Coverage) = -0.00602 ; sdMAF P-value = 0.157584761057514

Table BC: REF/ALT

	REF	ALT
Phase3 (GRCh37)	C	A
High Coverage	C	A

Table BD: male

	a	A	Total_P3
a	1182	0	1182
A	30	21	51
Total_HC	1212	21	1233

Table BE: female

	aa	Aa	AA	Total_P3
aa	911	1	0	912
Aa	333	25	0	358
AA	0	0	1	1
Total_HC	1244	26	1	1271

Note1. Row (Phase 3 GRCh37); Column (High Coverage GRCh38)
Note2. ‘a’: REF; ‘A’: ALT

Region: PAR3
rs number (GRCh37 phase3): rs113948071
SNP position (GRCh37) = 89803933 ; Female-Male Sex Difference in MAF (GRCh37 phase3) = 0.12154 ; sdMAF P-value = 2.45504806968182e-44
SNP position (GRCh38) = 90548934 ; Female-Male Sex Difference in MAF (High Coverage) = -0.000811 ; sdMAF P-value = 0.31711418273145

Table BF: REF/ALT

	REF	ALT
Phase3 (GRCh37)	A	C
High Coverage	A	C

Table BG: male

	a	A	Total_P3
a	1182	0	1182
A	50	1	51
Total_HC	1232	1	1233

Table BH: female

	aa	Aa	AA	Total_P3
aa	858	0	0	858
Aa	412	0	0	412
AA	1	0	0	1
Total_HC	1271	0	0	1271

Note1. Row (Phase 3 GRCh37); Column (High Coverage GRCh38)
Note2. ‘a’: REF; ‘A’: ALT

Region: PAR3
rs number (GRCh37 phase3): rs75914390
SNP position (GRCh37) = 90420559 ; Female-Male Sex Difference in MAF (GRCh37 phase3) = 0.13493 ; sdMAF P-value = 1.83772278515363e-34
SNP position (GRCh38) = 91165560 ; Female-Male Sex Difference in MAF (High Coverage) = 0.00849 ; sdMAF P-value = 0.308025448569295

Table BI: REF/ALT

	REF	ALT
Phase3 (GRCh37)	C	G
High Coverage	C	G

Table BJ: male

	a	A	Total_P3
a	1118	0	1118
A	43	72	115
Total_HC	1161	72	1233

Table BK: female

	aa	Aa	AA	Total_P3
aa	704	0	0	704
Aa	402	152	0	554
AA	1	6	6	13
Total_HC	1107	158	6	1271

Note1. Row (Phase 3 GRCh37); Column (High Coverage GRCh38)
Note2. ‘a’: REF; ‘A’: ALT

Region: PAR3
rs number (GRCh37 phase3): rs56118643
SNP position (GRCh37) = 90471772 ; Female-Male Sex Difference in MAF (GRCh37 phase3) = -0.0863 ; sdMAF P-value = 7.46065572919066e-08
SNP position (GRCh38) = 91216773 ; Female-Male Sex Difference in MAF (High Coverage) = -0.0031 ; sdMAF P-value = 0.853222915843523

Table BL: REF/ALT

	REF	ALT
Phase3 (GRCh37)	T	A
High Coverage	T	A

Table BM: male

	a	A	Total_P3
a	812	55	867
A	1	365	366
Total_HC	813	420	1233

Table BN: female

	aa	Aa	AA	Total_P3
aa	584	275	8	867
Aa	10	221	42	273
AA	0	0	131	131
Total_HC	594	496	181	1271

Note1. Row (Phase 3 GRCh37); Column (High Coverage GRCh38)
Note2. ‘a’: REF; ‘A’: ALT

Region: NPR
rs number (GRCh37 phase3): rs6637609
SNP position (GRCh37) = 128638559 ; Female-Male Sex Difference in MAF (GRCh37 phase3) = 0.2211 ; sdMAF P-value = 6.54681396294046e-63
SNP position (GRCh38) = 129504582 ; Female-Male Sex Difference in MAF (High Coverage) = 0.021 ; sdMAF P-value = 0.0770279808229025

Table BO: REF/ALT

	REF	ALT
Phase3 (GRCh37)	C	A
High Coverage	C	A

Table BP: male

	a	A	Total_P3
a	920	3	923
A	157	153	310
Total_HC	1077	156	1233

Table BQ: female

	aa	Aa	AA	Total_P3
aa	102	5	0	107
Aa	826	300	1	1127
AA	2	2	33	37
Total_HC	930	307	34	1271

Note1. Row (Phase 3 GRCh37); Column (High Coverage GRCh38)
Note2. ‘a’: REF; ‘A’: ALT

Region: PAR2
rs number (GRCh37 phase3): .
SNP position (GRCh37) = 154934428 ; Female-Male Sex Difference in MAF (GRCh37 phase3) = 0.3434 ; sdMAF P-value = 1.22828740905784e-169
SNP position (GRCh38) = 155704767 ; Female-Male Sex Difference in MAF (High Coverage) = 0.3442 ; sdMAF P-value = 5.16020258391841e-171

Table BR: REF/ALT

	REF	ALT
Phase3 (GRCh37)	T	G
High Coverage	T	G

Table BS: male

	aa	Aa	AA	Total_P3
aa	1	1	0	2
Aa	0	745	0	745
AA	0	0	486	486
Total_HC	1	746	486	1233

Table BT: female

	aa	Aa	AA	Total_P3
aa	578	0	0	578
Aa	0	489	0	489
AA	0	1	203	204
Total_HC	578	490	203	1271

Note1. Row (Phase 3 GRCh37); Column (High Coverage GRCh38)
Note2. ‘a’: REF; ‘A’: ALT

Region: PAR2
rs number (GRCh37 phase3): .
SNP position (GRCh37) = 154934986 ; Female-Male Sex Difference in MAF (GRCh37 phase3) = 0.3433 ; sdMAF P-value = 4.76760207367504e-169
SNP position (GRCh38) = 155705325 ; Female-Male Sex Difference in MAF (High Coverage) = 0.3458 ; sdMAF P-value = 5.65681734446808e-173

Table BU: REF/ALT

	REF	ALT
Phase3 (GRCh37)	G	A
High Coverage	G	A

Table BV: male

	aa	Aa	AA	Total_P3
aa	1	5	0	6
Aa	0	741	0	741
AA	0	0	486	486
Total_HC	1	746	486	1233

Table BW: female

	aa	Aa	AA	Total_P3
aa	578	1	0	579
Aa	2	489	0	491
AA	0	0	201	201
Total_HC	580	490	201	1271

Note1. Row (Phase 3 GRCh37); Column (High Coverage GRCh38)
Note2. ‘a’: REF; ‘A’: ALT

Region: PAR2
rs number (GRCh37 phase3): .
SNP position (GRCh37) = 154938547 ; Female-Male Sex Difference in MAF (GRCh37 phase3) = 0.343 ; sdMAF P-value = 2.93224328230306e-169
SNP position (GRCh38) = 155708886 ; Female-Male Sex Difference in MAF (High Coverage) = 0.3434 ; sdMAF P-value = 2.95168132842575e-169

Table BX: REF/ALT

	REF	ALT
Phase3 (GRCh37)	T	C
High Coverage	T	C

Table BY: male

	aa	Aa	AA	Total_P3
aa	2	1	0	3
Aa	0	744	0	744
AA	0	1	485	486
Total_HC	2	746	485	1233

Table BZ: female

	aa	Aa	AA	Total_P3
aa	577	0	0	577
Aa	3	486	2	491
AA	0	0	203	203
Total_HC	580	486	205	1271

Note1. Row (Phase 3 GRCh37); Column (High Coverage GRCh38)
Note2. ‘a’: REF; ‘A’: ALT

Region: PAR2
rs number (GRCh37 phase3): .
SNP position (GRCh37) = 154950587 ; Female-Male Sex Difference in MAF (GRCh37 phase3) = -0.4092 ; sdMAF P-value < 1e-300
SNP position (GRCh38) = 155720925 ; Female-Male Sex Difference in MAF (High Coverage) = -0.41 ; sdMAF P-value < 1e-300

Table CA: REF/ALT

	REF	ALT
Phase3 (GRCh37)	T	C
High Coverage	T	C

Table CB: male

	aa	Aa	AA	Total_P3
aa	21	1	0	22
Aa	0	953	1	954
AA	0	0	257	257
Total_HC	21	954	258	1233

Table CC: female

	aa	Aa	AA	Total_P3
aa	859	0	0	859
Aa	0	349	2	351
AA	0	2	59	61
Total_HC	859	351	61	1271

Note1. Row (Phase 3 GRCh37); Column (High Coverage GRCh38)
Note2. ‘a’: REF; ‘A’: ALT

Region: PAR2
rs number (GRCh37 phase3): .
SNP position (GRCh37) = 154954348 ; Female-Male Sex Difference in MAF (GRCh37 phase3) = -0.408 ; sdMAF P-value < 1e-300
SNP position (GRCh38) = 155724686 ; Female-Male Sex Difference in MAF (High Coverage) = -0.4084 ; sdMAF P-value < 1e-300

Table CD: REF/ALT

	REF	ALT
Phase3 (GRCh37)	C	T
High Coverage	C	T

Table CE: male

	aa	Aa	AA	Total_P3
aa	23	1	0	24
Aa	1	952	0	953
AA	0	0	256	256
Total_HC	24	953	256	1233

Table CF: female

	aa	Aa	AA	Total_P3
aa	859	0	0	859
Aa	0	351	0	351
AA	0	1	60	61
Total_HC	859	352	60	1271

Note1. Row (Phase 3 GRCh37); Column (High Coverage GRCh38)
Note2. ‘a’: REF; ‘A’: ALT

Region: PAR2
rs number (GRCh37 phase3): .
SNP position (GRCh37) = 154954591 ; Female-Male Sex Difference in MAF (GRCh37 phase3) = -0.408 ; sdMAF P-value < 1e-300
SNP position (GRCh38) = 155724929 ; Female-Male Sex Difference in MAF (High Coverage) = -0.4088 ; sdMAF P-value < 1e-300

Table CG: REF/ALT

	REF	ALT
Phase3 (GRCh37)	C	G
High Coverage	C	G

Table CH: male

	aa	Aa	AA	Total_P3
aa	22	1	0	23
Aa	0	952	0	952
AA	0	0	258	258
Total_HC	22	953	258	1233

Table CI: female

	aa	Aa	AA	Total_P3
aa	855	1	0	856
Aa	0	354	0	354
AA	0	2	59	61
Total_HC	855	357	59	1271

Note1. Row (Phase 3 GRCh37); Column (High Coverage GRCh38)
Note2. ‘a’: REF; ‘A’: ALT

Region: PAR2
rs number (GRCh37 phase3): .
SNP position (GRCh37) = 154957867 ; Female-Male Sex Difference in MAF (GRCh37 phase3) = -0.4088 ; sdMAF P-value < 1e-300
SNP position (GRCh38) = 155728205 ; Female-Male Sex Difference in MAF (High Coverage) = -0.4088 ; sdMAF P-value < 1e-300

Table CJ: REF/ALT

	REF	ALT
Phase3 (GRCh37)	C	A
High Coverage	C	A

Table CK: male

	aa	Aa	AA	Total_P3
aa	22	0	0	22
Aa	0	952	0	952
AA	0	0	259	259
Total_HC	22	952	259	1233

Table CL: female

	aa	Aa	AA	Total_P3
aa	857	1	0	858
Aa	0	350	0	350
AA	0	1	62	63
Total_HC	857	352	62	1271

Note1. Row (Phase 3 GRCh37); Column (High Coverage GRCh38)
Note2. ‘a’: REF; ‘A’: ALT

Region: PAR2
rs number (GRCh37 phase3): .
SNP position (GRCh37) = 154969038 ; Female-Male Sex Difference in MAF (GRCh37 phase3) = -0.3985 ; sdMAF P-value < 1e-300
SNP position (GRCh38) = 155739376 ; Female-Male Sex Difference in MAF (High Coverage) = -0.3973 ; sdMAF P-value < 1e-300

Table CM: REF/ALT

	REF	ALT
Phase3 (GRCh37)	T	C
High Coverage	T	C

Table CN: male

	aa	Aa	AA	Total_P3
aa	21	2	0	23
Aa	1	926	2	929
AA	0	0	281	281
Total_HC	22	928	283	1233

Table CO: female

	aa	Aa	AA	Total_P3
aa	812	4	0	816
Aa	0	384	2	386
AA	0	0	69	69
Total_HC	812	388	71	1271

Note1. Row (Phase 3 GRCh37); Column (High Coverage GRCh38)
Note2. ‘a’: REF; ‘A’: ALT

Region: PAR2
rs number (GRCh37 phase3): .
SNP position (GRCh37) = 154971425 ; Female-Male Sex Difference in MAF (GRCh37 phase3) = -0.3978 ; sdMAF P-value < 1e-300
SNP position (GRCh38) = 155741763 ; Female-Male Sex Difference in MAF (High Coverage) = -0.3974 ; sdMAF P-value < 1e-300

Table CP: REF/ALT

	REF	ALT
Phase3 (GRCh37)	A	C
High Coverage	A	C

Table CQ: male

	aa	Aa	AA	Total_P3
aa	37	0	0	37
Aa	0	931	1	932
AA	0	0	264	264
Total_HC	37	931	265	1233

Table CR: female

	aa	Aa	AA	Total_P3
aa	841	2	0	843
Aa	0	361	1	362
AA	0	1	65	66
Total_HC	841	364	66	1271

Note1. Row (Phase 3 GRCh37); Column (High Coverage GRCh38)
Note2. ‘a’: REF; ‘A’: ALT

Region: PAR2
rs number (GRCh37 phase3): .
SNP position (GRCh37) = 154977261 ; Female-Male Sex Difference in MAF (GRCh37 phase3) = -0.3584 ; sdMAF P-value = 3.6584742279397e-212
SNP position (GRCh38) = 155747599 ; Female-Male Sex Difference in MAF (High Coverage) = -0.3553 ; sdMAF P-value = 2.97098408770581e-205

Table CS: REF/ALT

	REF	ALT
Phase3 (GRCh37)	T	C
High Coverage	T	C

Table CT: male

	aa	Aa	AA	Total_P3
aa	28	0	0	28
Aa	2	830	14	846
AA	0	11	348	359
Total_HC	30	841	362	1233

Table CU: female

	aa	Aa	AA	Total_P3
aa	675	9	0	684
Aa	5	461	7	473
AA	0	2	112	114
Total_HC	680	472	119	1271

Note1. Row (Phase 3 GRCh37); Column (High Coverage GRCh38)
Note2. ‘a’: REF; ‘A’: ALT
