# Supplementary material for: Major sex differences in allele frequencies for X chromosomal variants in both the 1000 Genomes Project and gnomAD
Source: PLoS Genet. 2022 May 31;18(5):e1010231. doi: 10.1371/journal.pgen.1010231 (PMC9187127; doi:10.1371/journal.pgen.1010231)
Supplement: S3 Note — (PDF) [file pgen.1010231.s035.pdf]

Supplementary Note 3

Derivations of the theoretical bounds shown in the Bland-Altman plot for X chromosomal SNPs with different minor alleles between females and males.

The discussion is based on the fact that in Bland-Altman plot, the regions where dots (SNPs) can locate are bounded (It is true for any type of SNPs). These regions have straight line boundaries through coordinate (0,0) or (0.5,0), which are the target objects we analyze here.

In the following analysis, assume male sample size n_m, female sample size n_f, male allele frequency (AF, refers to minor allele in combined group) f_m, and female AF f_f.

1 SNPs with flipped minor allele between female and male

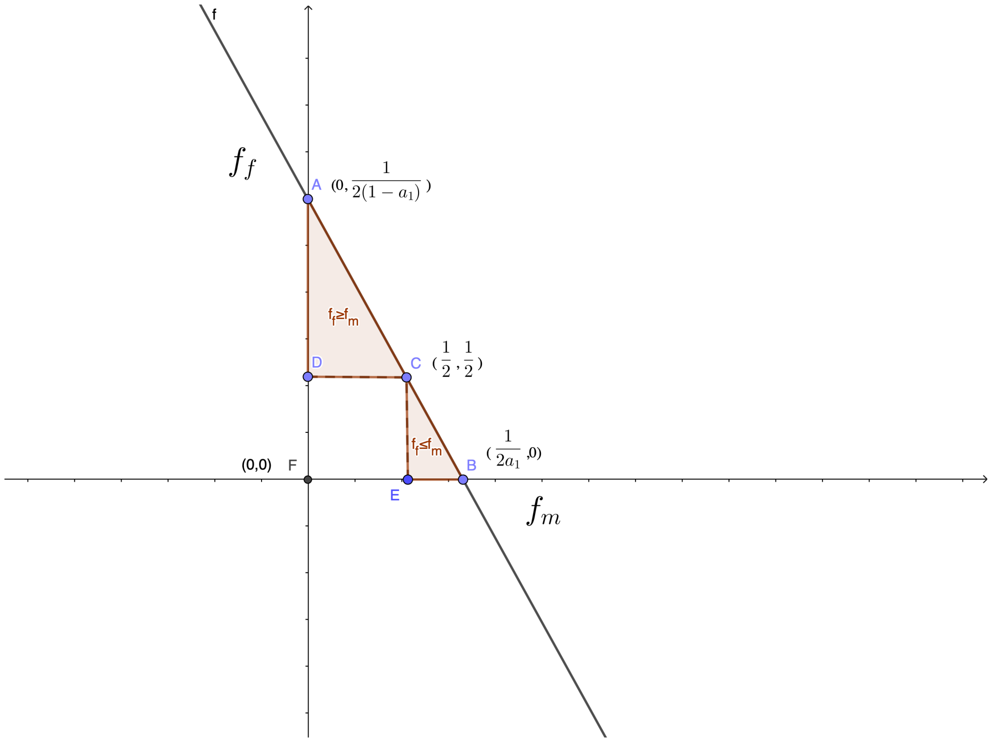

Figure A: Inequality constraint illustration.

Denote $a_1 = \frac{n_m}{n_m+2n_f}$ and $a_2 = \frac{n_m}{n_m+n_f}$.

For NPR & PAR3 SNPs:

Combined AF: $f_c = \frac{n_m f_m + 2n_f f_f}{n_m+2n_f} = a_1 f_m + (1-a_1) f_f$.

Since combined AF is always less or equal to 0.5, we have

$$f_f \le -\frac{a_1}{1-a_1} f_m + \frac{1}{2(1-a_1)}.$$

As shown in Figure 1, the solution space lies below line f. Since f_f and f_m are in the range $[0,1]$, the solution space where some point (f_m, f_f) can locate is within triangle AFB. This applies to all SNPs in NPR and PAR3 regions.

The slope of straight lines through coordinate $(0.5, 0)$ in Bland-Altman plot are given by

$$(f_f - f_m) \Big/ \left(\frac{n_m f_m + 2n_f f_f}{n_m + 2n_f} - \frac{1}{2} \right) = \frac{1}{1-a_1} - \frac{1}{(1-a_1)^2 (f_f - \frac{1}{2})/(f_m - \frac{1}{2}) + a_1(1-a_1)},$$

in which $\frac{f_f - 1/2}{f_m - 1/2}$ is the key term to analyze.

Note that $\frac{f_f - 1/2}{f_m - 1/2}$ is the slope of the line crossing point C in Figure 1. When $f_f \ge \frac{1}{2} \ge f_m$, the solution space is contained in triangle ADC, thus it holds that

$$\max \left\{ \frac{f_f - 1/2}{f_m - 1/2} \right\} = 0,$$
$$\min \left\{ \frac{f_f - 1/2}{f_m - 1/2} \right\} = -\frac{a_1}{1-a_1},$$

where maximum and minimum are achieved at line segments DC and AC. Since $(f_f - f_m) \Big/ \left(\frac{n_m f_m + 2n_f f_f}{n_m + 2n_f} - \frac{1}{2} \right)$ is monotone in $\frac{f_f - 1/2}{f_m - 1/2}$, by inserting these two values into the formula above, we can conclude that there are two boundary lines of slopes $-\frac{1}{a_1}$ and $-\infty$ (vertical line) crossing coordinate $(0.5, 0)$ in Bland-Altman plot for SNPs in NPR and PAR3 regions.

Similarly, when $f_f \le \frac{1}{2} \le f_m$, the solution space is contained in triangle CEB, thus it holds that

$$\max \left\{ \frac{f_f - 1/2}{f_m - 1/2} \right\} = -\frac{a_1}{1-a_1},$$
$$\min \left\{ \frac{f_f - 1/2}{f_m - 1/2} \right\} = -\infty,$$

where maximum and minimum are achieved at line segments CB and CE. Therefore, there are another two boundary lines of slopes $+\infty$ (vertical line) and $\frac{1}{1-a_1}$ crossing coordinate $(0.5, 0)$ in Bland-Altman plot for SNPs in NPR and PAR3 regions. Combined with the previous two boundary lines, the ultimate constraint region is composed of two conical regions intersecting at point $(0.5, 0)$.

For PAR1 & PAR2 SNPs:

Combined RAF: $f_c = \frac{n_m f_m + n_f f_f}{n_m + n_f} = a_2 f_m + (1-a_2) f_f$.

The solution space in Figure 1 for NPR and PAR3 SNPs can be directly applied to SNPs in PAR1 and PAR2 regions, with a_1 replaced by a_2.

The slope of straight lines through coordinate $(0.5,0)$ in Bland-Altman plot are given by

$$(f_f - f_m) \Big/ \left(\frac{n_m f_m + n_f f_f}{n_m + n_f} - \frac{1}{2}\right) = \frac{1}{1-a_2} - \frac{1}{(1-a_2)^2(f_f - \frac{1}{2})/(f_m - \frac{1}{2}) + a_2(1-a_2)}$$

It is trivial to see that we can replace a_1 with a_2 to obtain the parallel results for PAR1 and PAR2 SNPs. Therefore, there are three straight boundary lines of slopes $-\frac{1}{a_2}$, $\frac{1}{1-a_2}$ and ∞ crossing coordinate $(0.5,0)$ in Bland-Altman plot for SNPs in PAR1 and PAR2 regions, and the ultimate constraint region is composed of two conical regions intersecting at point $(0.5,0)$.

2 SNPs with the same minor allele between female and male

We can obtain similar results for SNPs with the same minor allele between female and male. In this case, the boundary lines in Bland-Altman plot are through coordinate $(0,0)$.

For NPR & PAR3 SNPs:

Combined AF: $f_c = \frac{n_m f_m + 2n_f f_f}{n_m + 2n_f} = a_1 f_m + (1-a_1) f_f$.

Note that the slope of boundary lines through coordinate $(0,0)$ in Bland-Altman plot is given by

$$(f_f - f_m)/f_c = (f_f - f_m) \Big/ \left(\frac{n_m f_m + 2n_f f_f}{n_m + 2n_f}\right) = \frac{1}{1-a_1} - \frac{1}{(1-a_1)^2 f_f/f_m + a_1(1-a_1)}.$$

Since combined AF is always less or equal to 0.5, we have

$$f_f \leq -\frac{a_1}{1-a_1} f_m + \frac{1}{2(1-a_1)}.$$

As shown in Figure 1, the solution space lies below function line f. Since f_f and f_m are in the range $[0,1]$, the solution space for (f_m, f_f) is contained in triangle AFB.

For points in triangle AFB, we have

$$\max\left\{(f_f - f_m) \Big/ \left(\frac{n_m f_m + 2n_f f_f}{n_m + 2n_f}\right)\right\} = \frac{1}{1-a_1},$$
$$\min\left\{(f_f - f_m) \Big/ \left(\frac{n_m f_m + 2n_f f_f}{n_m + 2n_f}\right)\right\} = -\frac{1}{a_1},$$

where maximum and minimum are achieved at line segments AF and BF. Recall that x-axis and y-axis in Bland-Altman plot refer to combined AF and sdMAF respectively, thus the above results indicate that there are two straight boundary lines of slopes $-\frac{1}{a_1}$ and $\frac{1}{1-a_1}$ crossing coordinate $(0,0)$ in Bland-Altman plot for SNPs in NPR and PAR3 regions.

For PAR1 & PAR2 SNPs:

Combined RAF: $f_c = \frac{n_m f_m + n_f f_f}{n_m + n_f} = a_2 f_m + (1-a_2) f_f$.

The slope of boundary lines through coordinate $(0,0)$ in Bland-Altman plot is given by $(f_f - f_m)/f_c = (f_f - f_m) \Big/ \left(\frac{n_m f_m + n_f f_f}{n_m + n_f}\right) = \frac{1}{1-a_2} - \frac{1}{(1-a_2)^2 f_f/f_m + a_2(1-a_2)}$.

It is trivial to see that we can replace a_1 with a_2 to obtain the parallel results for PAR1 and PAR2 SNPs. Therefore, there are two straight boundary lines of slopes $-\frac{1}{a_2}$ and $\frac{1}{1-a_2}$ crossing coordinate $(0,0)$ in Bland-Altman plot for SNPs in PAR1 and PAR2 regions.
