# Supplementary material for: Major sex differences in allele frequencies for X chromosomal variants in both the 1000 Genomes Project and gnomAD
Source: PLoS Genet. 2022 May 31;18(5):e1010231. doi: 10.1371/journal.pgen.1010231 (PMC9187127; doi:10.1371/journal.pgen.1010231)
Supplement: Attachment — Submitted filename: response-to-reviewers(2).docx [file pgen.1010231.s041.docx]

**Response to Reviewer #1**

The authors report X-chromosome allele frequency differences in 1000 Genomes data. The data are of interest, but the statistical analyses do not take into account the methodology of Graffelman (Heredity 116:558-568, 2016; Genetic Epidemiology 42:34-48, 2018).

**Response:** We were aware of Graffelman and Weir (2016; Testing for Hardy-Weinberg equilibrium at biallelic genetic markers on the X chromosome) but cited (reference #23 in the original submission) their follow-up work of Graffelman, Jain and Weir (2017; A genome-wide study of Hardy-Weinberg equilibrium with next generation sequence data), in which “X-chromosomal variants were tested by an omnibus exact test for HWP in females and equality of allele frequencies in males and females (Graffelman and Weir 2016)”; we have added the reference of Graffelman and Weir (2016) to the revised manuscript.

The original submission contained the following discussion: *Hardy-Weinberg equilibrium testing of variants in the 1000 Genomes phase 3 data has been examined separately in the JPT and YRI populations (23). SNPs with missing rate <5% and possessing an rs identifier were used. The earlier work (23) found lower rates of deviation from HWE on the X chromosome (after exclusion of PAR1 and PAR2) than on any of the autosomes, but the sample size for the X chromosome is ~3/4 of that for the autosomes. Results from our sdMAF analysis also calls for new X chromosome-aware HWE methods that consider sdMAF.*

The original submission also contained a detailed HWE analyses for top ranked SNPs, stratified by the five super-populations; see section “ In-depth analysis of eight SNPs with the most significant sdMAF in the phase 3 data” and results in Dataset S1 for additional details.

Except for the above discussion, we did not comment further on the connection between sdMAF and HWE for two reasons. First, we did not want to distract the readers from the main message of sdMAF. Second, as noted in our 2021 ASHG abstract (Zhang et al. PrgmNr 3161: A novel regression-based method for X-chromosome-inclusive Hardy-Weinberg equilibrium test), “Graffelman and Weir (2016) proposed a 2 d.f. test that includes the deviation of male genotype counts from the expected based on pooled allele frequency estimate using both male and female data. We show that the 2 d.f. Pearson’s Chisq test of Graffelman and Weir (2016) can be reformulated as simultaneously testing sex differences in allele frequency [sdMAF], and HWE in female group alone.” Because of this confounding between HWE and sdMAF testing, we felt that the X chromosome HWE testing method of Graffelman and Weir (2016) needs additional evaluation as our future work.

Thank you for directing our attention to Graffelman and Weir (2018), which focused on *autosomal* biallelic SNPs. Graffelman and Weir (2018) proposed and evaluated methods that test equality of allele frequencies between sexes (EAF; sdMAF in our notation) and Hardy-Weinberg proportions (HWP; testing for HWD by convention) either jointly or separately using a likelihood ratio (LR)-based approach. They considered six (A–F) scenarios, and “In order to determine which scenario best describes a marker, successive hypothesis tests can be performed until a model is found that cannot be rejected.” Additionally, “among the models that cannot be rejected, the one that has fewer parameters” is favored and “model selection can also be performed by calculating Akaike's information criterion (AIC; Akaike, 1973), for all six models and choosing the model with the smallest AIC.”

Graffelman and Weir (2018) then compared the LR-based approach with an omnibus exact test, that tests for sdMAF and HWD simultaneously, and “this approach has been used by Graffelman and Weir (2016) to test biallelic variants on the X chromosome for HWP…Figure 3 shows that the omnibus exact test [of Graffelman and Weir (2016)] has a better Type I error rate than the LR test [of Graffelman and Weir (2018)]. [Specifically,] for low MAF variants, the LR test is more conservative than the exact test, and for higher MAF, the LR test is above the nominal level.”

Thus, the approach in Graffelman and Weir (2016) appears to be superior to that in Graffelman and Weir (2018). However, as noted earlier, the 2 d.f. test of Graffelman and Weir (2016) is jointly testing for sdMAF and HWD using both females and males. Additionally, their 2 d.f. test can be partitioned into two 1 d.f. tests, one for sdMAF testing using both females and males, and the other for HWD testing in females (Zhang et al., 2021), as we have done here to avoid confounding sdMAF with HWD.

We have revised the manuscript to provide more justification of our approach.

**Response to Reviewer #2**

**General comments:**

In the manuscript ‘Major sex differences in allele frequencies for X chromosome variants in the 1000 Genomes Project data’, the authors examine sex differences in minor allele frequency on the X chromosome using 1000 genomes project data, focusing on the different X chromosome regions, comparisons between low and high coverage sequencing, and comparisons to the autosomes. They observe sex differences in allele frequency in PAR1 and PAR2, which may point to underlying biology and differences likely due to linkage, whereas differences in the NPR and PAR3 are likely due to genotyping errors in lower coverage data. The paper is extremely well-written and clear, and represents an important contribution, considering that the X chromosome is often excluded from analyses because of a lack of understanding of the complex issues and appropriate resources and analysis tools. These results have implications for how X chromosome data should be analyzed and interpreted, and notably also points to interesting biology that may underly the observed differences in variant frequencies between males and females. Specific observations and clarifications are outlined below.

**Response:** We thank the reviewer’s appreciation of our work. We have numbered the reviewer's comments and suggestions, and we provide a point-to-point response below.

**Specific comments:**

**#1.** The investigation breaks out SNPs in region, between non-PAR and PAR. But what about SNPs in the non-PAR region that reside within genes that have homologous copies on the Y? What do the allele frequencies look like for these SNPs on both the X and the Y in males? Similarly, what do the allele frequencies look like on the Y more generally? Perhaps this would also serve as an appropriate comparison, especially for the PAR region.

**Response:** Using biomart from ensembl build 38, we identified 19 genes that have homologous copies in the non-PAR regions of both X and Y; see the newly added Supplementary Dataset S3. In total, 52 SNPs from these genes have sdMAF p-value < 5e-8 in the phase 3 data. We have included this information in the revised manuscript, including a newly added Supplementary Dataset S4.

Further examination of allele frequencies of the Y chromosome is interesting but beyond the scope of this work. For the SNPs in the PAR regions in particular, understanding the allele frequencies of the Y also requires accurate phasing information. We have added this discussion to the revised manuscript.

**#2.** How big are the observed sex differences in MAF? The focus is mostly on p-values, but what are the general distributions of the effect size differences? These are shown in figures, but only described for highly significant variants.

**Response:** Part (B) of Figures 2-5 provide the “Female-Male sdMAF for the same SNPs in part A [which shows the p-values]”, and Figure 6 shows the “Bland-Altman plots comparing the Female-Male sex difference in MAF to the sex-combined MAF across the X chromosome”, stratified by the four regions. We have revised the corresponding text to highlight this information.

Additionally, we now include the following summary statistics in the revised manuscript: *Among the analyzed SNPs, 0.39%, 16.88%, 1.54%, and 1.18% of the SNPs have absolute(sdMAF)>=0.05, respectively, in PAR1, PAR2, PAR3, and NPR regions. For these SNPs, the medians of absolute(sdMAF) are respectively 0.095, 0.121, 0.074, and 0.077, and the [Q1,Q3] are [0.060,0.274], [0.064,0.286], [0.060,0.099], and [0.059,0.111], respectively, in PAR1, PAR2, PAR3, and NPR regions.*

**#3.** The in-depth analysis of 8 SNPs shows some interesting patterns. How wide-spread are these across the significant variants? For example, minor allele flips between males and females, and the fixed homozygous genotype in females but fixed heterozygous in males (or mix of homozygous and heterozygous in the less extreme case). This suggests that one allele is fixed on the X and the other allele is fixed on the Y. How is it possible in the PAR with recombination between X and Y? Because it is an A/T, is this actually due to an error/strand flip issue? Or is the recombination rate 0 in this region?

**Response:** Among the 2,039 SNPs with genome-wide significant sdMAF, 66 (3.24%) have minor allele flips between males and females. We have included this information in the revised manuscript.

Additionally, we added the following supplementary figure (on the next page) to show the “minor allele flips between males and females” X chromosome-wide, *without requiring* the sdMAF testing to be genome-wide significant. Among this set of SNPs, only one SNP in PAR2 (POS=154941870) have close to sex-specific genotypes: A1A1=0, A1A2=20, A2A2=1251 in females, and A1A1=9, A1A2=1219, A2A2=5 in males.

As this SNP has also genotype-wide significant sdMAF, the above genotype counts information also appears in the newly included Supplementary Dataset S2, which is similar to Dataset S1 but includes all 2,039 SNPs with genome-wide significant sdMAF.

Finally, it is unlikely that strand flips are a major cause of the difference in genotype by sex, since variant calling was performed blind to sex and typically joint-called, as opposed to being called on an individual basis, especially for the high coverage 1000 genomes data (Marta et al. 2021) and the newly included gnomAD data (Karczewski et al. 2020). Further, inspection of Integrative Genomics Viewer (IGV) plots in gnomAD v3.1.2 shows that there are few discrepancies of sequencing reads around these SNPs, making it unlikely that they are strand flips. We have added this discussion to the revised manuscript.

**Bland-Altman plots for X chromosomal SNPs *with different minor alleles between females and males* from the 1000 Genomes Project phase 3 data on GRCh37.** Regions are plotted separately A: NPR; B: PAR1, C: PAR2; D: PAR3. For each of the four regions, the histogram at the top of the Bland-Altman plot shows the distribution of the sex-combined MAF for bi-allelic SNPs with global MAF ≥5% presumed to be of high quality. The histogram to the right of the plot shows the distribution of the Female - Male sdMAF. The red dotted lines are the theoretical bounds; see Supplementary Note S3 for deviations.

**#4.** Why do we see higher rates of allele frequency differences in PAR2 compared to other regions, particularly PAR1? Is this in line with the higher recombination rate (overall and sex-specific) observed in PAR1 in Monteiro et al?

**Response:** We believe that it is in line with the recombination rate observation made in Monteiro et al. (2021), but future work is needed to test this hypothesis as the consistent observations could be due to the fact that some of the same data were used for both analyses. In addition, PAR2 is smaller than PAR1.

In response to Reviewer 3’s suggestion on performing a population-specific sdMAF analysis, we observed a similarly higher rate of sdMAF for PAR2 than PAR1 across the five super-populations; see the newly added figures (numbered as Figure S4-S8 in the revised manuscript), which show (A) the Manhattan plots for sdMAF testing and (B) the Female - Male sdMAF, respectively, for the AFR, AMR, EAS, EUR, and SAS super-populations.

We additionally studied sdMAF in the gnomAD data in the revised manuscript, and we observed similar patterns in both the non-Finnish European and African/African American super populations. Please see response to question #7 below for details of the sdMAF analysis of the gnomAD data.

**#5**. Why weren’t all SNPs in phase 3 examined in high-coverage data? Does the large lift-over failure rate point to systematic errors in the successful SNPs, or only the unsuccessful? Was the lift-over failure rate the same across the four regions?

**Response:** To compare the sdMAF results between the two datasets for the top ranked SNPs, we had to limit the SNPs that could be successfully lifted over. We have clarified this in the revised manuscript.

Since the initial submission of this manuscript, the version 1 of the biorxiv paper (Marta 2021) on the high-coverage data has been updated to version 2, which no longer mentions the X chromosome-wide lift-over failure rate from build 37 to build 38 for the phase 3 data, as this data has now been withdrawn as noted in <https://ftp.1000genomes.ebi.ac.uk/vol1/ftp/release/20130502/supporting/GRCh38_positions/README_GRCh38_liftover_20170504.txt>. We have therefore removed the lift-over-failure-rate statements in our revised manuscript.

Additionally, we performed a separate analysis of the high-coverage X chromosome-wide data *without* the lift-over restriction. The revised manuscript now has a new subsection in the Results section called “High coverage 1000 genomes X chromosome-wide sdMAF”, to make it clear this separate analysis of the high-coverage data without the lift-over restriction.

Specifically, Table S3 shows the counts of X chromosome variants by regions, global MAF, and types from the high coverage data of the 1000 Genomes Project on GRCh38. Figure S19 shows the analytical pipeline of the high coverage data, *without* the liftover restriction. Figures S20-S23 show (A) the Manhattan plots for sdMAF testing and (B) the Female - Male sdMAF, respectively, for the whole X chromosome, and PAR1, PAR2 and PAR3 regions.

**#6.** Did you consider a sliding window approach to compute allele frequencies and allele frequency differences? This may allow for a better regional description of patterns.

**Response:** Thank you for this very interesting suggestion. Focusing on the phase 3 data, we considered overlapping sliding windows of 50 SNPs with a step of 25 SNPs. We then took the average of the -log10 p-values, and provided the result as a Supplementary figure as shown below. Ten regions remain genome-wide significant including one each in PAR1, PAR2 and PAR3, occurring at the PAR-NPR boundaries as in the single-SNP analysis, and seven in NPR. We have added this discussion to the revised manuscript.

**Manhattan plot of sdMAF *sliding window* p-values across the X chromosome from the 1000 Genomes Project phase 3 data on GRCh37.** Each window contains 50 adjacent bi-allelic SNPs (with global MAF ≥5%) and are moved by 25 SNPs each time. A sliding window sdMAF p-value (on the –log10 scale) is the average of –log10 p-value of the 50 SNPs in the window. The position of each window is represented by the position of the leftmost SNP. SNPs in the PAR1, PAR2 and PAR3 regions are plotted in grey, with PAR3 located around 90 Mb. Y-axis is $-$log10(sdMAF p-values) and p-values >0.1 are plotted as 0.1 (1 on $-$log10 scale) for better visualization. The dashed red line represents 5e-8 (7.3 on the $-$log10 scale).

**#7.1** Are similar patterns seen in other datasets, e.g. HRC and TopMed? And are sex differences in allele frequency seen with rare variants?

**Response:** Thank you for this suggestion. As sex-specific MAFs are unavailable for either the HRC and TopMed data, but they are available for the gnomAD data, we thus analyzed the sdMAF in the gnomAD data. We focused on the non-Finnish European sample, and separately the African/African American sample; these two super populations have the largest sample sizes in the gnomAD data.

In short, similar sdMAF were observed in the gnomAD data for SNPs with MAF>5%; see the newly added two figures below, which are similar to Figure 2 for the sdMAF analysis of the 1000 Genomes project data. The details of this new analysis can be found in the newly added Method section called “gnomAD v3.1.2” and results are presented in the newly added section called “High-coverage whole genome sequence from gnomAD v3.1.2” in the revised manuscript.

The non-Finnish European sample of gnomAD The African/African American sample of gnomAD

**Manhattan plot for testing for sex difference in MAF across the X chromosome of the (Left) Non-Finnish European population and (Right) the African/African American population from the gnomAD v3.1.2 data on GRCh38.** A: sdMAF p-values for bi-allelic SNPs with MAF ≥5%, presumed to be of high quality. SNPs in the PAR1, PAR2 and PAR3 regions are plotted in grey, with PAR3 located around 90 Mb. Y-axis is $-$log10(sdMAF p-values) and p-values >0.1 are plotted as 0.1 (1 on $-$log10 scale) for better visualization. The dashed red line represents 5e-8 (7.3 on the $-$log10 scale). B: Female - Male sdMAF for the same SNPs in part A.

**#7.2** And are sex differences in allele frequency seen with rare variants?

**Response:** We performed a sdMAF analysis focusing on variants with MAF in the range of 0.1% <MAF <5% using the non-Finnish European sample of gnomAD. For a rare variant with MAF=0.1%, we expect to see about 50 copies of the minor allele given the large sample size; sdMAF testing of variants with MAF <0.1% would require developing an appropriate exact test that is beyond the scope of our study here. The revised manuscript includes the following supplementary figure, which shows that sdMAF is observed for rare variants.

**Manhattan plot for testing for sex difference in MAF, for SNPs with 0.1%<MAF<5%, across the X chromosome of the Non-Finnish European population from the gnomAD v3.1.2 data on GRCh38.** A: sdMAF p-values for bi-allelic SNPs with 0.1%<MAF<5%. SNPs in the PAR1, PAR2 and PAR3 regions are plotted in grey, with PAR3 located around 90 Mb. Y-axis is $-$log10(sdMAF p-values) and p-values >0.1 are plotted as 0.1 (1 on $-$log10 scale) for better visualization. The dashed red line represents 5e-8 (7.3 on the $-$log10 scale). B: Female - Male sdMAF for the same SNPs in part A.

**#8.** It would be helpful to emphasize more the implications of these findings for analysis methods, especially for the PAR genes. For example, differences in allele frequency will lead to variance differences. What are the solutions and recommendations, and do they differ between PAR and non-PAR? Are sex-specific results necessary? Findings also seem to suggest that sex-specific QC is critical for the X (MAF, genotyping error, and HWE). Due to sex-specific differences in HWE, should this be part of QC?

**Response:** Indeed, these are all very important research questions on how to best analyze the available X chromosome data. Providing solutions to these questions is beyond the scope of this work, but the revised manuscript now discusses the implications of sdMAF for QC and association analysis, as well as possible next steps when appropriate. Please see response to comment #9 below for additional details.

**#9.** A paragraph on next steps, limitations, and/or conclusion

**Response:** Thank you for the suggestion, which we have incorporated into the revised manuscript.

**Response to Reviewer #3**

Review of “Major sex differences in allele frequencies for X chromosome variants in the 1000 Genomes Project data” by Wang et al.

This article reports the interesting finding that a set of SNPs on the X chromosome, in particular those close to the boundaries of the PAR regions, shows significant differences in allele frequency between males and females. The article has extensive supplementary graphics, reflecting results of considerable amount of data analysis performed with the samples from the 1000G project. I have some major and minor concerns detailed below.

**Response:** We thank the reviewer’s appreciation of our work. We have numbered the reviewer's comments and suggestions, and we provide a point-to-point response below.

**Major concerns:**

**#1.** In the introduction the author sketch that the X chromosome has been largely ignored in association studies, and that methods to deal with X chromosomal data have only recently been developed. This is certainly true, but not only so for association studies. In fact, even methods for testing for Hardy-Weinberg equilibrium at X chromosomal variants are very new (Graffelman & Weir, 2016). To my eyes the Introduction section of the paper is somewhat short, and a bit more background on the X chromosome (Wise et al., 2013) and the PAR regions (Graves et al., 1998) could strengthen the article.

**Response:** Following the reviewer’s comment, we have substantially expanded the Introduction section, as well as the Discussion section, in the revised manuscript. We have also added the Graffelman and Weir, 2016, and Graves et al., 1998 papers to the references; the work of Wise et al., 2013 was cited in the initial submission.

Please see responses to comment 4 below as well as response to Reviewer #1 about Graffelman & Weir (2016) and the related HWE testing.

Please see response to Reviewer #2’s comments 8 and 9 on the implications of sdMAF for QC and association analysis, and future research directions.

**#2.** The initial analysis on detecting sdMAF SNPs is based on the full 1000G data. This comprises individuals from 26 human populations and five continental regions worldwide. It is well known that allele frequencies of SNPs tend to vary over human populations, and that ignoring such population substructure can lead to spurious statistical associations in many types of analysis: Hardy-Weinberg equilibrium, linkage disequilibrium (LD) and association testing among others. In the Online methods section, the authors state in this regard about their sdMAF SNP test procedure: “This more powerful approach (i.e. by joining the populations) can detect sex difference in MAF if it is present in any of the sub-samples”. This is not quite true. If two populations both have a sex difference in MAF, but of opposite sign (e.g. population 1 having A as the minor allele, and population 2 having B as the minor allele) then joining those two populations averages out the difference in MAF between the sexes. This shows that joining samples from different populations is not always beneficial, but can lead to a loss of power to detect sdMAF SNPs. In order to avoid the effects of population substructure, one could just try to detect sdMAF SNPs WITHIN each population. That would probably give a fairer idea of how many of such sdMAF SNPs are around, and it could detect additional sdMAF SNPs that have may have gone unnoticed in the current analysis.

**Response:** Thank you for the suggestion. Indeed, our original plan for the analysis was population-specific but decided against it as because a) the type I error is controlled (slightly conservative) using the whole-sample analysis, b) SNPs with genome-wide significant sdMAF were observed across the X chromosome even using our potentially less powerful test (e.g. due to opposite direction of sdMAF as astutely pointed out by the reviewer); that is, our primary research goal is pointing out the sdMAF issue, not developing the most powerful sdMAF test, and c) We thought the population-specific analysis for the SNPs with the most significant sdMAF (Dataset S1 and Section “In-depth analysis of eight SNPs with the most significant sdMAF in the phase 3 data“) convey the message more concisely.

Nevertheless, we performed the population-specific analysis as suggested. In addition to the superpopulation-specific Manhattan plot, the revised manuscript also provides the following scatter plot, contrasting the sdMAF estimates between the populations, which show remarkable consistency.

**Matrix of pairwise scatter plots for comparing sdMAF between combined sample (ALL) and each of the five superpopulations from the 1000 Genomes Project phase 3 data on GRCh37.** The bi-alleic SNPs shown are the ones with global MAF ≥5% in the combined ALL sample, polymorphic in each of the five superpopulations, and genome-wide significant in at least one of the six sdMAF analyses (i.e. in ALL or any of the five superpopulations). Both X-axis and Y-axis are sdMAF. The dashed line represents locations where X and Y have the same sdMAF. Two solid grey lines represent locations where sdMAF from either group are zeros. Each dot in the scatter plots is colored based on the position of the corresponding SNP, with SNPs from PAR1, PAR2, PAR3 and NPR regions colored blue, yellow, red and black respectively.

In addition to contrasting the sdMAF estimates, the revised manuscript includes the following scatter plot (on the next page), contrasting the sdMAF p-values (on the -log10 scale) for SNPs with genome-wide significant sdMAF in at least one of the six sdMAF analyses (i.e. in ALL or any of the five superpopulations).

The top row of the figure on the next page shows that the combined analysis is overall much more powerful than the superpopulation-specific analysis, identifying many SNPs with genome-wide significant sdMAF that would be missed by the individual superpopulation analysis, and often with sdMAF p-values several magnitudes smaller.

In contrast, although the individual superpopulation analysis may identify some SNPs with genome-wide significant sdMAF that would be missed by the proposed ALL analysis (see the newly added table below), the sdMAF p-values are comparable as shown in the scatter plots on the next page, top row of the figure.

**Contrasting combined sample (ALL) and superpopulation-specific analysis from the 1000 Genomes Project phase 3 data on GRCh37.** The numbers of SNPs with genome-wide significant sdMAF in the superpopulation-specific analysis but not in the ALL analysis, stratified by the superpopulations and regions. The nTotal is the non-overlapping total, stratified by the four regions.

**Matrix of pairwise scatter plots for comparing sdMAF p-values between combined sample (ALL) and each of the five superpopulations from the 1000 Genomes Project phase 3 data on GRCh37.** The bi-alleic SNPs shown are the ones with global MAF ≥5% in the combined ALL sample, polymorphic in each of the five superpopulations, and genome-wide significant in at least one of the six sdMAF analyses (i.e. in ALL or any of the five superpopulations). Both X-axis and Y-axis are $-$log10(sdMAF p-values) and log-scaled for better visualization. The dashed line is the main diagonal line. Each dot is colored based on the position of the corresponding SNP, with SNPs from PAR1, PAR2, PAR3, and NPR regions colored blue, yellow, red, and black, respectively.

Additionally, we performed a meta-analysis and the revised manuscript includes the following Miami plot:

**Miami plot for sdMAF p-values obtained from mega-analysis (top) and meta-analysis (bottom) across the X chromosome from the 1000 Genomes Project phase 3 data on GRCh37.** The bi-alleic SNPs shown are the ones with global MAF ≥5% in the combined sample, and polymorphic in each of the five superpopulations (for meta-analysis to have consistent sample sizes across SNPs). SNPs in the PAR1, PAR2 and PAR3 regions are plotted in grey, with PAR3 located around 90 Mb. Y-axis is $-$log10(sdMAF p-values) and p-values >0.1 are plotted as 0.1 (1 on $-$log10 scale) for better visualization. The dashed red line represents 5e-8 (7.3 on the $-$log10 scale). For meta-analysis, a SNP’s allele can be sex-specific in a superpopulation. For example, A1 is present only in females, and A2 is present only in males (MAF could be still greater than 5%). In that case, the sdMAF test statistic would be infinity due to variance being zero in the superpopulation sdMAF analysis, which leads to meta-analysis p-value being zero. In those cases, the p-values are plotted as 47,982.36 on the –log10 scale, the smallest p-value observed in the mega-analysis.

Finally, as suggested by Reviewer 2 in comment 7, we performed a separate sdMAF analysis using the gnomAD data. We focused on the non-Finnish European sample, and separately the African/African American sample; these two super-populations have the largest sample sizes in the gnomAD data. We observed similar sdMAF patterns as in the 1000 Genomes Project data. The details of this new analysis can be found in the newly added Method section called “gnomAD v3.1.2” and results are presented in the newly added section called “High-coverage whole genome sequence from gnomAD v3.1.2” in the revised manuscript.

**#3.** The authors use a fixed genome-wide significance level of 5E-8 throughout, for all chromosomes studied, but the question arises if this is adequate. They find 6, 1 and 0 sdMAF SNPs on the autosomes 1, 7 and 22 respectively, but such decay is to be expected, simply because the larger chromosomes have more SNPs, and thus also more significant ones. Whether there is truly a different rate of significant variants across the chromosomes is probably better judged by comparing the number of significant results after a Bonferroni correction for each chromosome, since this will take the number of SNPs on each chromosome into account.

**Response:** As suggested by the reviewer, we have revised the manuscript to clarify that we did not imply “there is truly a different rate of significant variants across the chromosomes”. We have added a sentence to emphasize that the difference is between the X chromosome and autosomes analyzed.

**#4.1** The authors conduct tests for HWE of X chromosomal variants. They use the standard autosomal asymptotic chi-square test for the NPR and PAR3 region, using the females only. This is not to be recommend for various reasons. First of all, the HWE exact test is the state-of-the-art statistical methodology for testing HWE, for having in general, a better type 1 error rate and power (Wigginton et al., 2005; Graffelman & Moreno, 2013). Moreover, by running the test on females only, the male X chromosomal alleles are ignored, leading to loss of power, and, importantly, if male and female allele frequencies differ HWE will not hold due to the fact that convergence to equilibrium can take several generations for the X chromosome (Crow & Kimura, 1970), and this can go undetected by testing females only. The best way to test the NPR (and presumably, the PAR3 region, which is not really established as pseudo-autosomal) is to use the X chromosomal exact test for HWE, specially developed for this purpose by Graffelman & Weir (2016).

**Response:** We have added the references of Wigginton et al. (2005), Graffelman & Moreno (2013), and Crow & Kimura (1970) to our revised manuscript.

Reviewer 1 also made a comment on the use of the HWE testing method of Graffelman & Weir (2016), and in our response we stated that

As noted in our 2021 ASHG abstract (Zhang et al. PrgmNr 3161: A novel regression-based method for X-chromosome-inclusive Hardy-Weinberg equilibrium test), “Graffelman and Weir (2016) proposed a 2 d.f. test that includes the deviation of male genotype counts from the expected based on pooled allele frequency estimate using both male and female data. We show that the 2 d.f. Pearson’s Chisq test of Graffelman and Weir (2016) can be reformulated as simultaneously testing sex differences in allele frequency [sdMAF], and HWE in female group alone.” Because of this confounding between HWE and sdMAF testing, we felt that the X chromosome HWE testing method of Graffelman and Weir (2016) needs additional evaluation as our future work.

**#4.2** For testing variants in PAR1, the authors report p-values can be “NA” for monomorphic variants. This again refers to asymptotic testing with the Chi-square test. The exact test for HWE can deal with monomorphic variants, and yields a p-value of 1 for monomorphic variants, which looks more neat.

**Response:** Thank you for this suggestion. We have added the exact HWE test p-value to the revised Dataset S1 as column V, the last column of the Excel sheet.

**#4.3** For PAR1 and PAR2, the natural thing is to use the standard autosomal exact test for HWE for all observations (males and females), and given the focus of the paper on sdMAF SNPs, one might consider to do the exact test also for males and females separately. However, it has shown that tests for equal allele frequencies in the sexes and for HWE are intricately linked in their assumptions (Graffelman & Weir, 2018), and therefore, for PAR1 and PAR2 the joint exact test for equality of allele frequencies and Hardy-Weinberg proportions is indicated. The forgoing comments show that the “new X chromosome-aware HWE methods that consider sdMAFs” the authors call for do in fact already exist but they were apparently not noticed by the authors.

**Response:** Reviewer 1 also made a comment on the work of Graffelman & Weir (2018), and in our response we stated that

Thank you for directing our attention to Graffelman and Weir (2018), which focused on autosomal biallelic SNPs. Graffelman and Weir (2018) proposed and evaluated methods that test equality of allele frequencies between sexes (EAF; sdMAF in our notation) and Hardy-Weinberg proportions (HWP; testing for HWD by convention) either jointly or separately using a likelihood ratio (LR)-based approach. They considered six (A–F) scenarios, and “In order to determine which scenario best describes a marker, successive hypothesis tests can be performed until a model is found that cannot be rejected.” Additionally, “among the models that cannot be rejected, the one that has fewer parameters” is favored and “model selection can also be performed by calculating Akaike's information criterion (AIC; Akaike, 1973), for all six models and choosing the model with the smallest AIC.”

Graffelman and Weir (2018) then compared the LR-based approach with an omnibus exact test, that tests for sdMAF and HWD simultaneously, and “this approach has been used by Graffelman and Weir (2016) to test biallelic variants on the X chromosome for HWP…Figure 3 shows that the omnibus exact test [of Graffelman and Weir (2016)] has a better Type I error rate than the LR test [of Graffelman and Weir (2018)]. [Specifically,] for low MAF variants, the LR test is more conservative than the exact test, and for higher MAF, the LR test is above the nominal level.”

Thus, the approach in Graffelman and Weir (2016) appears to be superior to that in Graffelman and Weir (2018). However, as noted earlier, the 2 d.f. test of Graffelman and Weir (2016) is jointly testing for sdMAF and HWD using both females and males. Additionally, their 2 d.f. test can be partitioned into two 1 d.f. tests, one for sdMAF testing using both females and males, and the other for HWD testing in females (Zhang et al., 2021), as we have done here to avoid confounding sdMAF with HWD.

Finally, Graffelman and Weir (2018) also showed examples (from an application to autosomal SNPs using the n=104 individuals from the JPT sample of the 1000 Genomes Project) that, in the presence of sdMAF, true HWD may go unnoticed if applying the standard HWD testing using both males and females.

**Minor issues:**

**#5.** “X chromosome variants” in the title probably better worded as “X chromosomal variants”.
I suggest to replace 245825 SNPs by 245825 X-chromosomal SNPs to stress the number is specific for the X chromosome.

**Response:** Thank you for the careful reading and constructive comment. We have revised the manuscript accordingly.
