# Supplementary material for: Major sex differences in allele frequencies for X chromosomal variants in both the 1000 Genomes Project and gnomAD
Source: PLoS Genet. 2022 May 31;18(5):e1010231. doi: 10.1371/journal.pgen.1010231 (PMC9187127; doi:10.1371/journal.pgen.1010231)
Supplement: Attachment — Submitted filename: R2-response-to-reviewer(2).docx [file pgen.1010231.s042.docx]

**Round 2 reviewers and responses**

**Reviewer #1**

The authors have addressed my concerns.

**Reviewer #2**

My previous concerns have all been addressed. I have no further comments.

**Response to Reviewer #3**

Re-review of “Major sex differences in allele frequencies for X chromosome variants in the 1000 Genomes Project data” by Wang et al.

This article has improved after the first round of review. However, the authors fail to adequately address several of the issues previously expressed. I have the following remaining major and minor concerns. The comments below refer to the updated manuscript, using line numbers of the new clean version without tracked changes.

**Response:** We have numbered the reviewer's additional comments and suggestions, and we provide a point-to-point response below.

Major concerns:

**#1.** References. The authors claim to “have also added the …. Graves et al., 1998 paper to the references”, however the truth is that they have not cited Graves et al. (1998) in the new version of the manuscript.

**Response:** We sincerely apologize for this inadvertent omission. Graves et al. (1998) is now included in the revised manuscript.

**#2.** L182: with respect to the in-depth analysis of a small set of most significant sdMAF SNPs, the paper may benefit from a table, included in the main manuscript, containing genotype counts and test results for sdMAF and HWE of these SNPs. This avoids the reader has to browse supplementary Excel files in order to see some detailed examples of, after all, the most salient sdMAF SNPs, which are the core topic of this paper.

**Response:** We have done this as suggested (thank you), and we have added the following Table as a main display.

**[Newly added] Table 1.** SNP name is from gnomAD which incorporates chromosome-nucleotide position and reference-alternate alleles; Population NFE=non-Finnish European; AFR=African/African American. Sex: F=female, M=male; call rate is proportion of individuals with non-missing genotypes; RR= reference homozygote/hemizygote genotype count; RA=heterozygote genotype count; AA=alternate allele homozygote/hemizygote genotype count. sdMAF is female-male allele frequency difference, along with p value. HWD delta is the Hardy-Weinberg equilibrium delta, along with p value, NA=not applicable.

| **X chr Region** | **SNP (GRCh38)** | **gnomAD Population** | **Sex** | **Call rate (%)** | **RR** | **RA** | **AA** | **Minor Allele** | **sdMAF** | **sdMAF.p** | **HWD.delta** | **HWE.p** |
| --- | --- | --- | --- | --- | --- | --- | --- | --- | --- | --- | --- | --- |
| **PAR1** | **X-2779827-G-A** | **NFE** | **F** | **99.72** | **19436** | **194** | **1** | **A** |  |  | **0.000** | **0.46** |
| **PAR1** | **X-2779827-G-A** | **NFE** | **M** | **99.50** | **626** | **13577** | **67** | **A** | **-0.475** | **<1E-300** | **-0.226** | **<1E-300** |
| **PAR1** | **X-2779827-G-A** | **AFR** | **F** | **99.16** | **6772** | **3707** | **514** | **A** |  |  | **0.000** | **0.82** |
| **PAR1** | **X-2779827-G-A** | **AFR** | **M** | **99.08** | **248** | **7346** | **1975** | **A** | **-0.375** | **<1E-300** | **-0.142** | **<1E-300** |
| **PAR1** | **X-2780882-G-A** | **NFE** | **F** | **99.85** | **19567** | **89** | **0** | **A** |  |  | **0.000** | **0.75** |
| **PAR1** | **X-2780882-G-A** | **NFE** | **M** | **99.64** | **91** | **14150** | **39** | **A** | **-0.496** | **<1E-300** | **-0.245** | **<1E-300** |
| **PAR1** | **X-2780882-G-A** | **AFR** | **F** | **99.51** | **8781** | **2118** | **133** | **A** |  |  | **0.000** | **0.68** |
| **PAR1** | **X-2780882-G-A** | **AFR** | **M** | **99.64** | **137** | **8442** | **1044** | **A** | **-0.439** | **<1E-300** | **-0.191** | **<1E-300** |
| **NPR** | **X-52861869-T-C** | **NFE** | **F** | **98.54** | **14176** | **5222** | **1** | **C** |  |  | **-0.018** | **1.45E-103** |
| **NPR** | **X-52861869-T-C** | **NFE** | **M** | **85.50** | **12189** | **NA** | **73** | **C** | **0.129** | **<1E-300** | **NA** | **NA** |
| **NPR** | **X-52861869-T-C** | **AFR** | **F** | **99.36** | **9642** | **1368** | **5** | **C** |  |  | **-0.003** | **6.00E-10** |
| **NPR** | **X-52861869-T-C** | **AFR** | **M** | **90.36** | **8552** | **NA** | **175** | **C** | **0.042** | **1.43E-84** | **NA** | **NA** |
| **PAR3** | **X-89688092-A-G** | **NFE** | **F** | **99.48** | **13994** | **5106** | **483** | **G** |  |  | **0.001** | **0.50** |
| **PAR3** | **X-89688092-A-G** | **NFE** | **M** | **89.78** | **11538** | **NA** | **1338** | **G** | **0.051** | **1.37E-55** | **NA** | **NA** |
| **PAR3** | **X-89688092-A-G** | **AFR** | **F** | **99.56** | **9694** | **1300** | **43** | **G** |  |  | **0.000** | **0.93** |
| **PAR3** | **X-89688092-A-G** | **AFR** | **M** | **84.52** | **7865** | **NA** | **298** | **G** | **0.026** | **2.44E-23** | **NA** | **NA** |
| **PAR3** | **X-90958827-T-C** | **NFE** | **F** | **99.38** | **15016** | **4218** | **330** | **C** |  |  | **0.001** | **0.09** |
| **PAR3** | **X-90958827-T-C** | **NFE** | **M** | **91.08** | **12039** | **NA** | **1025** | **C** | **0.046** | **1.66E-57** | **NA** | **NA** |
| **PAR3** | **X-90958827-T-C** | **AFR** | **F** | **99.31** | **9611** | **1353** | **45** | **C** |  |  | **0.000** | **0.72** |
| **PAR3** | **X-90958827-T-C** | **AFR** | **M** | **85.60** | **7990** | **NA** | **277** | **C** | **0.032** | **3.17E-35** | **NA** | **NA** |
| **NPR** | **X-141051591-A-G** | **NFE** | **F** | **99.31** | **14846** | **4700** | **5** | **G** |  |  | **-0.014** | **6.07E-79** |
| **NPR** | **X-141051591-A-G** | **NFE** | **M** | **79.08** | **11253** | **NA** | **89** | **G** | **0.113** | **<1E-300** | **NA** | **NA** |
| **NPR** | **X-141051591-A-G** | **AFR** | **F** | **98.57** | **8826** | **2095** | **7** | **G** |  |  | **-0.009** | **2.57E-25** |
| **NPR** | **X-141051591-A-G** | **AFR** | **M** | **84.68** | **8057** | **NA** | **121** | **G** | **0.082** | **<1E-300** | **NA** | **NA** |
| **PAR2** | **X-155706522-G-A** | **NFE** | **F** | **98.23** | **19321** | **16** | **0** | **A** |  |  | **0.000** | **0.95** |
| **PAR2** | **X-155706522-G-A** | **NFE** | **M** | **97.92** | **30** | **14012** | **2** | **A** | **-0.499** | **<1E-300** | **-0.249** | **<1E-300** |
| **PAR2** | **X-155706522-G-A** | **AFR** | **F** | **98.71** | **10933** | **10** | **0** | **A** |  |  | **0.000** | **0.96** |
| **PAR2** | **X-155706522-G-A** | **AFR** | **M** | **99.26** | **11** | **9573** | **3** | **A** | **-0.499** | **<1E-300** | **-0.249** | **<1E-300** |
| **PAR2** | **X-155712209-T-G** | **NFE** | **F** | **99.63** | **19585** | **28** | **0** | **G** |  |  | **0.000** | **0.92** |
| **PAR2** | **X-155712209-T-G** | **NFE** | **M** | **99.12** | **29** | **14186** | **2** | **G** | **-0.498** | **<1E-300** | **-0.249** | **<1E-300** |
| **PAR2** | **X-155712209-T-G** | **AFR** | **F** | **99.58** | **10520** | **516** | **3** | **G** |  |  | **0.000** | **0.19** |
| **PAR2** | **X-155712209-T-G** | **AFR** | **M** | **99.78** | **14** | **9404** | **218** | **G** | **-0.487** | **<1E-300** | **-0.238** | **<1E-300** |

**#3.** Population substructure. The way population substructure is currently addressed in the paper leaves still much to desire. In the first place the authors “account for” population substructure by doing separate analyses at the CONTINENTAL level. Allele frequency differences over SNPs within continents are certainly known to exist. The authors never truly go down to testing at the population level. Repeating the analysis for all 26 1000G populations is of course a prodigious amount of work, and not really required, but evidence for the existence of sdMAF SNPs would certainly be more convincing, if all 26 populations are tested for at least some of the most salient sdMAF SNPs that are found by the authors. This will be insightful, because it will also show if sdMAF SNPs can be population specific, or if they are preserved across all populations. Moreover, as pointed out in the first review, testing within each population may detect additional sdMAF SNPs that have may have gone unnoticed in the continental analysis, and this could at least be mentioned.

The limitations of the sdMAF test (such as the mentioned loss of power due to the averaging out of allele frequency differences across populations in the previous review) should be clearly addressed in the Discussion section, in particular because no detailed population-level analysis has been carried out.

**Response:** In the previous revision, we added

**Table S2. Contrasting combined sample (ALL) and super-population-specific analysis from the 1000 Genomes Project phase 3 data on GRCh37.** The numbers of SNPs with genome-wide significant sdMAF in the super-population-specific analysis but not in the ALL analysis, stratified by the super-populations and regions. The nTotal is the non-overlapping total, stratified by the four regions.

We noted that there is a remarkable consistency in sdMAF estimates between the five super-populations as shown in Figure S10, which was included in the previous revision but included here for the ease of reading. Thus, we believe results would be similar between the 26 populations.

Nevertheless, we have added additional analysis as suggested by the reviewer, and revised the statement as:

*In contrast, although the individual super-population (continent-stratified) analysis identified some SNPs with significant sdMAF that would be missed by the ALL analysis (Table S2), the sdMAF p values are comparable as shown in Figure S9. There is also a remarkable consistency in sdMAF estimates between the five super populations as shown in Figure S10. We also performed a meta-analysis with sample-size based weighting of all five super-populations and compared the results to the primary ALL analysis (above). Figure S11 (Miami) and Figure S12 (pp plot) show that the results are consistent between the two analyses.*

*Although unlikely, it is possible that the above continent-stratified analysis may fail to identify sdMAF SNPs with heterogeneity (in terms of signs of the sdMAF estimates) in sdMAF between populations. Thus, for the eight X chromosomal SNPs with the most salient results (as initially discussed in Dataset S1) from the 1000 Genomes phase 3 data, we further conducted a population-stratified sdMAF analysis, separately for each of the 26 populations.*

*Figure 3 shows that, as expected, the sdMAF results for these SNPs are consistent across the 26 populations. For example, for rs6634333 (POS=140993859) from the NPR region (Figure 3B), the sdMAF estimate in the overall sample is 0.338 (p-value=3.78E-151). While the 26 population-specific estimates are not identical, all 26 estimates are positive and range from 0.206 (p-value=0.011) in STU to 0.443 (p-value=2.90E-9) in ESN (Supplementary Dataset S5). Remarkable consistencies across populations are also observed for the other seven SNPs.*

***Figure 3. Population-specific sdMAF results for 8 X chromosomal SNPs from 1000 Genomes Project phase 3 data across the 26 populations.*** *The 8 SNPs were selected (two each from the 4 regions: PAR1, PAR2, PAR3 and NPR) with the smallest sdMAF p values (see text). Analysis was performed separately for the 26 populations and sdMAF (female-male, blue circles) as well as their corresponding -log_10_(p value) (black triangles) are plotted on the left and right Y axes, respectively. Populations are labeled on the X axis using the conventional 1000 Genomes Project codes, and grouped by super-population. Physical position is GRCh37 and rs provided where available. The red dashed horizontal line is a p=5x10^-8^. Part A shows results for PAR1 and PAR2, Part B for PAR3 and NPR.*

**#4.** Hardy-Weinberg equilibrium testing. On lines 214-219 the authors refer to HWE testing in the PAR region, and their comment that “monomorphic females can have p-value=NA” shows they continue to focus on the chi-square test, whereas exact procedures are known to be more powerful. As already pointed out in my previous review: “The exact test for HWE can deal with monomorphic variants, and yields a p-value of 1 for monomorphic variants, which looks more neat. For PAR1 and PAR2, the natural thing is to use the standard autosomal exact test for HWE”. The authors should clearly mention the aptness of the classical exact test for the PAR regions, and state that monomorphic SNPs will not have p-value NA but p-value 1 in that case.

**Response:** We note that the Exact test was included in the previous revision. For example, the last column (HWExact.p) of Supplementary Datasets 1 and 2 included the p-values of the Exact test. On the general merits of the Exact test, please see the response to question #5.2 below.

**#5.1** On lines 415-416 the authors state “Earlier work has examined HWD of X chromosomal variants (58, 59), using a 2 degrees of freedom (df) test that jointly analyses both females and males…”. This sentence is notoriously false. Reference 59, Graffelman et al. (2017), clearly and explicitly state all SNPs (autosomal and X-chromosomal) are tested with EXACT tests, NOT with a 2df chi-square test. The exact tests are to be preferred, because they are the most powerful and state-of-the-art approach to HWE testing. Moreover, Graffelman and Weir (2016) explicitly show the Type 1 error rate of the Exact test for X is better than the 2DF chi-square test for X.

**Response:** We now only cite (59), Graffelman and Weir (2016), in this statement. We cite (58), Graffelman et al. (2017), elsewhere when discussing the EXACT vs. chisq-based test; please see below.

**#5.2.** On lines 422-424 the authors state “And for NPR and PAR3 variants, we performed the HWD analysis in females only; the males have two hemizygous genotypes, leaving no df to perform the HWD test after using one df to estimate the MAF in males.” First of all, this is the most common situation on X, as the non-recombining NPR part is the largest, and contains most of the SNPs. Second, what the authors do is to apply the classical one-degree-of-freedom chi-square test for the females only in this case. That is not the best choice of test. There is no need to exclude the male alleles. As explicitly argued by Graffelman & Weir (2016), for NPR (and PAR3) X chromosomal variants, a specific X-chromosomal exact test can be used that uses ALL alleles (males and females), and this test is to be preferred for the reasons given above.

Most notoriously, the authors cite the article of Graffelman & Weir (2016), they analyse X chromosomal NPR/PAR3 data, but they do not even mention that a specific HWE Exact test procedure for NPR and PAR3 variants exists.

Consequently, the paragraph starting at line 415 should be completely revised.

On lines 565-570 the authors again stress the use of asymptotic chi-square-statistic for also doing the testing of HWE in females only in the NPR and PAR3 region. Again, in this case the most powerful test is the X-chromosomal Exact test for HWE; as already literally stated in the previous review “The best way to test the NPR (and presumably, the PAR3 region, which is not really established as pseudo-autosomal) is to use the X chromosomal exact test for HWE, specially developed for this purpose by Graffelman & Weir (2016).”

If the authors desire also to test females only, then they should just do the standard autosomal Exact test with the female genotype counts only. In the literature, this has been shown to be superior to the chi-square test; the exact test better controls the Type I error rate, and has better power (Wigginton, 2005; Graffelman & Moreno, 2013).

**Response:** The central theme of Graffelman & Weir (2016) is “In this paper we show that the males are relevant, because a difference in allele frequency between males and females [sdMAF] may indicate HWE not to hold.”

As discussed in round 1 revision (Response to Reviewer 1 and to Reviewer 3 questions #1 and #4), “Because of this confounding between HWE and sdMAF testing, we felt that the X chromosome HWE testing method of Graffelman and Weir (2016) needs additional evaluation as our future work.”

This delineation of sdMAF and HWE testing is particularly important because significant sdMAF can be biologically expected due to, for example, sex-linkage at the NPR-PAR boundaries.

In the presence of true sdMAF, a sex-combined HWE analysis may lead to increased type I error rate, which we provide specific examples below after the discussion of the EXACT test of Graffelman & Weir (2016).

Indeed, Graffelman & Weir (2016) developed “four frequentist statistical test procedures for X-linked markers that take both males and females into account: the χ2 test, likelihood ratio test, exact test and permutation test. Exact tests that include males are shown to have a better Type I error rate”. And Graffelman et al. (2017) applied the EXACT test to both autosomal and X chromosomal SNPs.

However, although the EXACT test does not specify the degrees of freedom, our preliminary work suggests that it is similar to the chisq test with 2 d.f. in spirit, because the EXACT test includes males and is derived conditional on “number of A alleles (nA) and the observed male and female frequencies.” This will lead to increased type I error HWE test in presence of true sdMAF.

For example, we used the R program HardyWeinberg v1.7.4 (https://cran.r-project.org/web/packages/HardyWeinberg/index.html), the EXACT test of Graffelman & Weir (2016), to analyze the following two illustrative SNPs.

- a NPR SNP, significant sdMAF but no deviation from HWE in females.
  - female MAF=0.2 with genotype counts (640, 320, 40)
  - male	MAF=0.5 with genotype counts (500, NA, 500)
  - sex-combined EXACT test result is highly significant due to sdMAF

> HWExact(c(AA=640,AB=320,BB=40,A=500,B=500),x.linked = T)

Graffelman-Weir exact test for Hardy-Weinberg equilibrium on the X-chromosome

using SELOME p-value

Sample probability 1.712993e-63 p-value = 6.418765e-61

- a PAR SNP, significant sdMAF but no deviation from HWE in females, and no deviation from HWE in males; the minor allele is defined in the sex-pooled sample.
  - female MAF=0.2 with genotype counts (640, 320, 40)
  - male	MAF=0.8 with genotype counts (40, 320, 640)
  - sex-combined EXACT test result is highly significant due to sdMAF

> HWExact(c(AA=640+40,AB=320+320,BB=40+640),x.linked = F)

Haldane Exact test for Hardy-Weinberg equilibrium (autosomal)

using SELOME p-value

sample counts: nAA = 680 nAB = 640 nBB = 680

H0: HWE (D==0), H1: D <> 0

D = -180 p-value = 0.00000000000000000000000000000000000000000000000000000000001501401

Indeed, the above results are consistent with those of Graffelman & Weir (2016) in their application study of “4158 SNPs at the X chromosome of the venous thrombosis database”. When discussing their Figure 6, the authors of Graffelman & Weir (2016) noted that “SNPs that become significant upon changing from a females-only test to an all-individual test typically have significant differences in male and female allele frequencies”.

Additionally, in their application, Graffelman & Weir (2016) noted that “The χ2 and exact P-values correlate well (plots not shown), and particularly so if low MAF markers are excluded.” We also note that the scale for -log10 (HWE P) is 0-6 in Figure 6 (b) for χ2 and 0-3 in Figure 6(d) for the EXACT test, suggesting that EXACT may not be more powerful than the chisq-based test.

Finally, we were not able to use HardyWeinberg v1.7.4 to analyze all gnomAD SNPs. For example, we received the following error message when analyzing rs12010572 (POS:3386942) of the SNP using the non-Finnish European female sample.

HardyWeinberg::HWExact(c(AA=4957,AB=9824,BB=4876),x.linked = F)

Error: C stack usage 7954064 is too close to the limit

These empirical results, combined with our (current) better analytical understanding of the 2 d.f. chi-square test of Graffelman & Weir (2016), led to our initial decision to focus on the discussion of the 2 d.f. test: As noted in our 2021 ASHG abstract (Zhang et al. PrgmNr 3161: A novel regression-based method for X-chromosome-inclusive Hardy-Weinberg equilibrium test), “We show that the 2 d.f. Pearson’s Chisq test of Graffelman and Weir (2016) can be reformulated as simultaneously testing sex differences in allele frequency [sdMAF], and HWE in female group alone.”

We have revised our manuscript to add a bit more discussion on the EXACT test. The revised discussion now reads:

*Earlier work has examined HWD of X chromosomal variants (59)* [Graffelman & Weir (2016)]*, using a 2 degrees of freedom (df) Pearson Chisq-based test that jointly analyzes both females and males. The test includes, in addition to the female data, the deviation of male genotype counts from the expected, based on pooled allele frequency estimate using both male and female data. However, it has been shown that this 2 df HWD test is equivalent to testing for HWD in females alone and simultaneously testing for sdMAF between the sexes (60). Because of this confounding between HWD and sdMAF, for variants in the PAR1 and PAR2 regions, we performed the HWD analysis stratified by the sex. And for NPR and PAR3 variants, we performed the HWD analysis in females only; the males have two hemizygous genotypes, leaving no df to perform the HWD test after using one df to estimate the MAF in males.*

*In addition to the 2 df Chisq test, the work of (59)* [Graffelman & Weir (2016)] *also proposed and recommended an Exact test, jointly analyzing both females and males. Although the EXACT test does not specify the degrees of freedom, our preliminary work suggests that it is similar to the 2 df Chisq test in spirit, because the Exact test includes males and is derived conditional on the “number of A alleles (nA) and the observed male and female frequencies.” This could lead to increased type I error HWD test in the presence of true sdMAF. For example, consider a SNP from the NPR region with significant sdMAF but no deviation from HWE in females: female MAF=0.2 with genotype counts of (640, 320, 40) and male MAF=0.5 with genotype counts of (500, NA, 500). In that case, the sex-combined EXACT test p=6.41E-61 (using the R program HardyWeinberg v1.7.4 (61)), but this highly significant Exact HWD testing result is due to the significant sdMAF alone. When the HWD analysis is performed using only females and in the presence of small sample, the Exact test can be advantageous (58, 59)* [Graffelman et al. (2017), Graffelman & Weir (2016)]

**#6.** L572: the reformulation in Eq. (9) is cited to be “recent work”, but is in fact well known, and much older, and can be found in, among others, Weir (1996).

**Response:** The reference of Weir (1996) was cited in the earlier submission but only in the Section – sdMAF test. As suggested, we now also cite it when discussing testing for HWD. The revised the statement now reads as

*We first note that earlier work (63) [Weir 1996] has shown that the Pearson’s chi-square-based HWD test can be reformulated as a test based on the HWD estimate, and more recent work (67) has shown that the same HWD test can be derived from a robust allele-based reverse regression.*

Minor issues:

L228: “have close” ◊ “has close”
L494: Table S3 seems to refer to table S4.
L512: “consists of” ◊ “consisting of”
L538: omit “with each other”
Equation (9): “r” is not defined, and seems mistaken, and apparently needs to be replaced by “f”.

**Response:** Thank you very much for the careful reading, and we have made the corresponding corrections in the revised manuscript. Yes, the “r” should be replaced by “f”. Thank you for catching this typo!

References:

Graves JAM, Wakefield MJ, Toder R (1998). The origin and evolution of the pseudoautosomal regions of human sex chromosomes. Human Molecular Genetics 7: 1991–1996. Doi: 10.1093/hmg/7.13.1991
Graffelman, J., Jain, D. and Weir, B.S. (2017) A genome-wide study of Hardy-Weinberg equilibrium with next generation sequence data. Human Genetics 136(6) pp. 727-741. doi: 10.1007/s00439-017-1786-7.
Weir, B. S. (1996) Genetic Data Analysis II. Sinauer Associates, Massachusetts.
Wigginton JE, Cutler DJ, Abecasis GR (2005). A note on exact tests of Hardy-Weinberg equilibrium. Am J Hum Genet 76: 887–893.
